# Supplementary material for: Genomic Analysis of Stropharia rugosoannulata Reveals Its Nutritional Strategy and Application Potential in Bioremediation
Source: J Fungi (Basel). 2022 Feb 6;8(2):162. doi: 10.3390/jof8020162 (PMC8874372; doi:10.3390/jof8020162)
Supplement: Supplementary file 1 [file jof-08-00162-s001.zip › Supplementary Material.pdf]

Supplementary material

Manuscript title: Genomic analysis of *Stropharia rugosoannulata* reveals the nutritional strategy and application potential in bioremediation

Information

1. Supplementary Figures 1 to 9: page 2-17

2. Supplementary Tables 1 to 18: page 18-54

Figure S1.

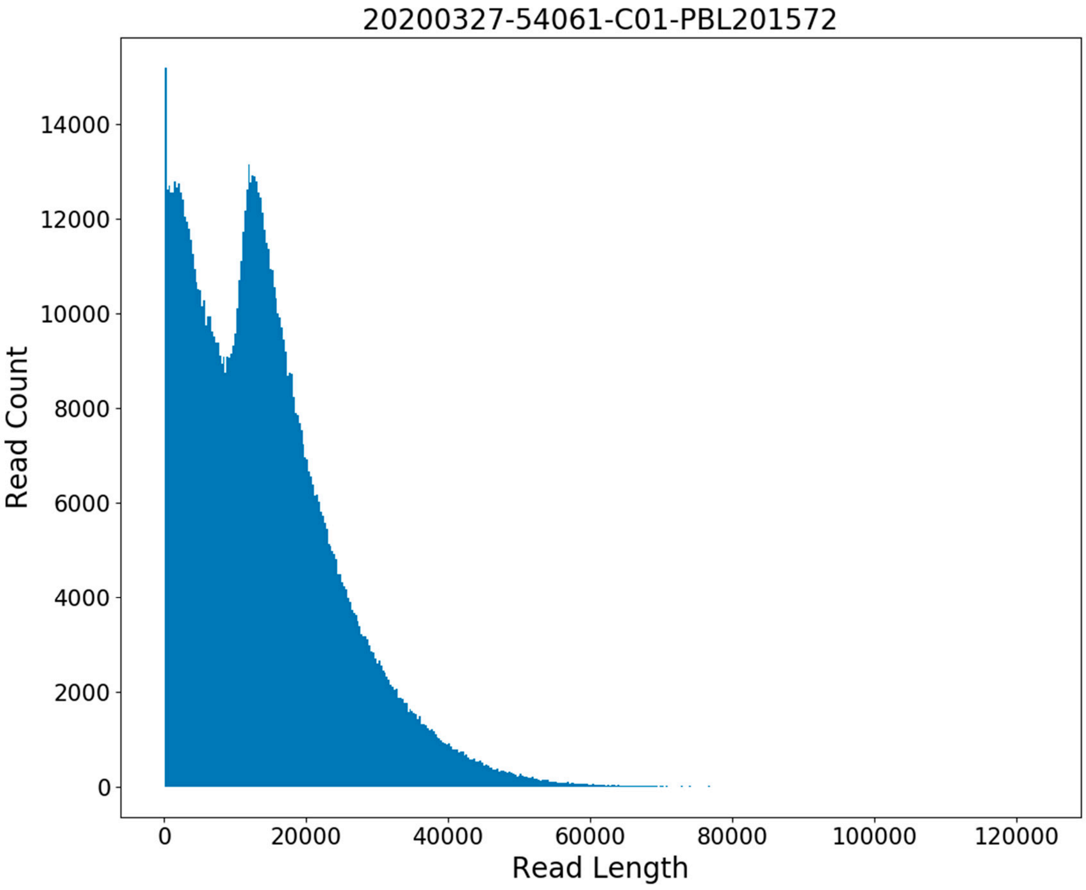

Figure S1. Statistical analysis for high-quality reads of the *Stropharia rugosoannulata* genome Abscissa and vertical represent the Subreads length, number of subread at the specified length, respectively. There is a total of 14.55 G clean data, 1,023,677 reads with an average read length of 14.21kb and the longest of 122.80Kb.

Figure S2.

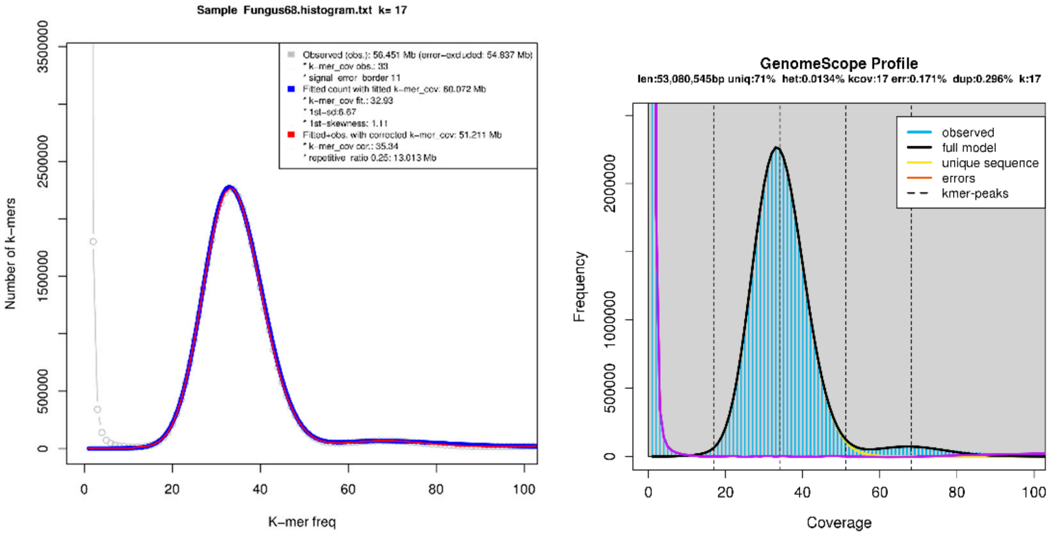

Figure S2. Heterozygosity analysis based on K-mer analysis

Figure S3.

A

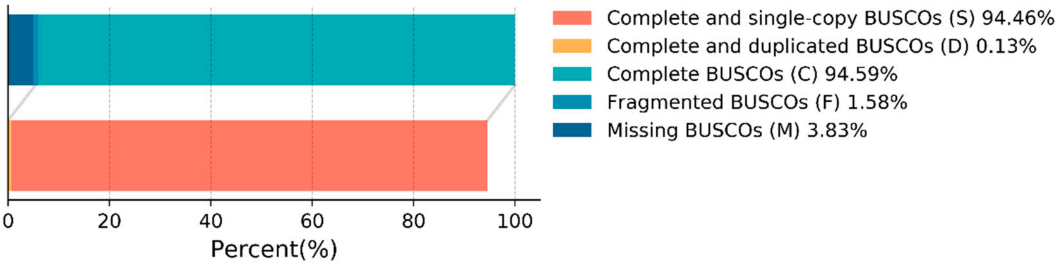

B

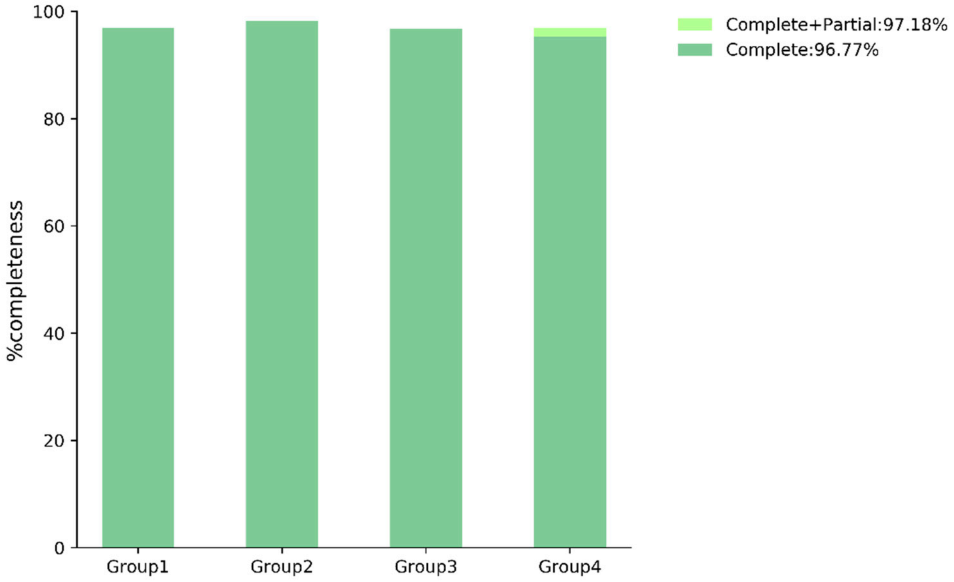

Figure S3. The completeness evaluation of the assembled genome

A BUSCO

B CEGMA

Figure S4.

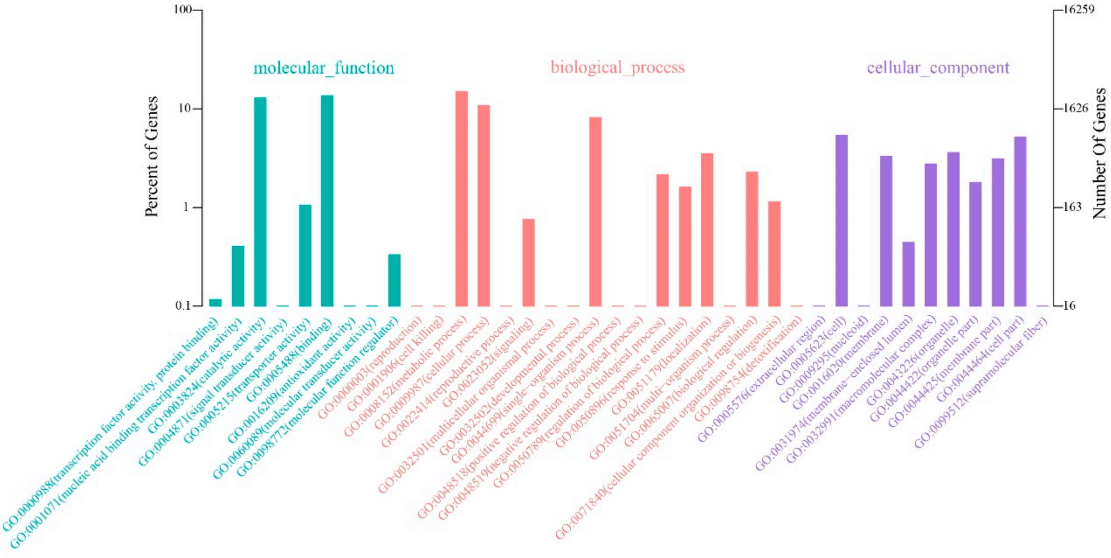

Figure S4. Go enrichment of predicted proteins in *Stropharia rogosoannulata*

Figure S5.

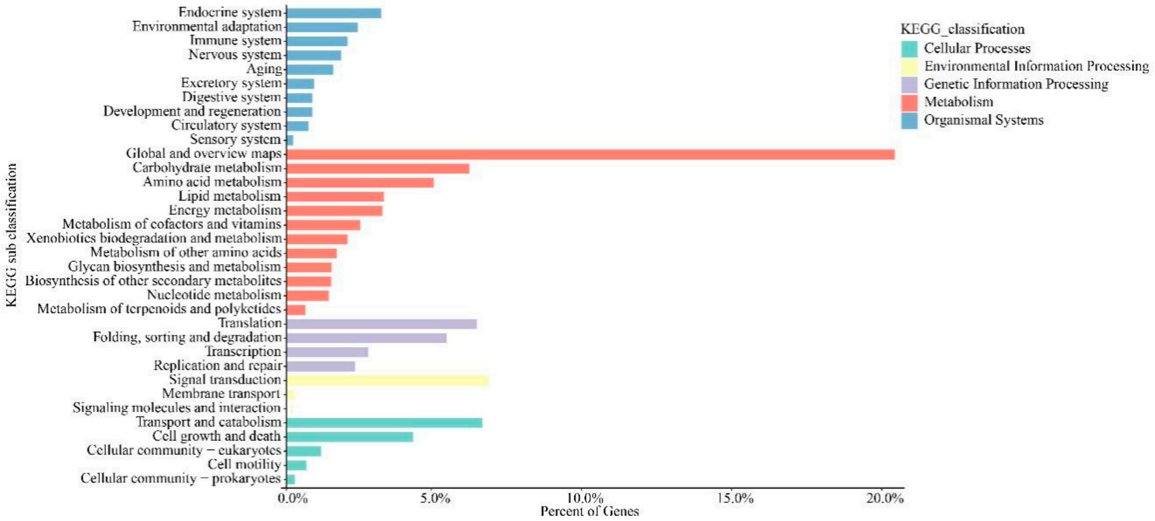

Figure S5. KEGG classification of predicted proteins in *Stropharia rogosoannulata*

Figure S6.

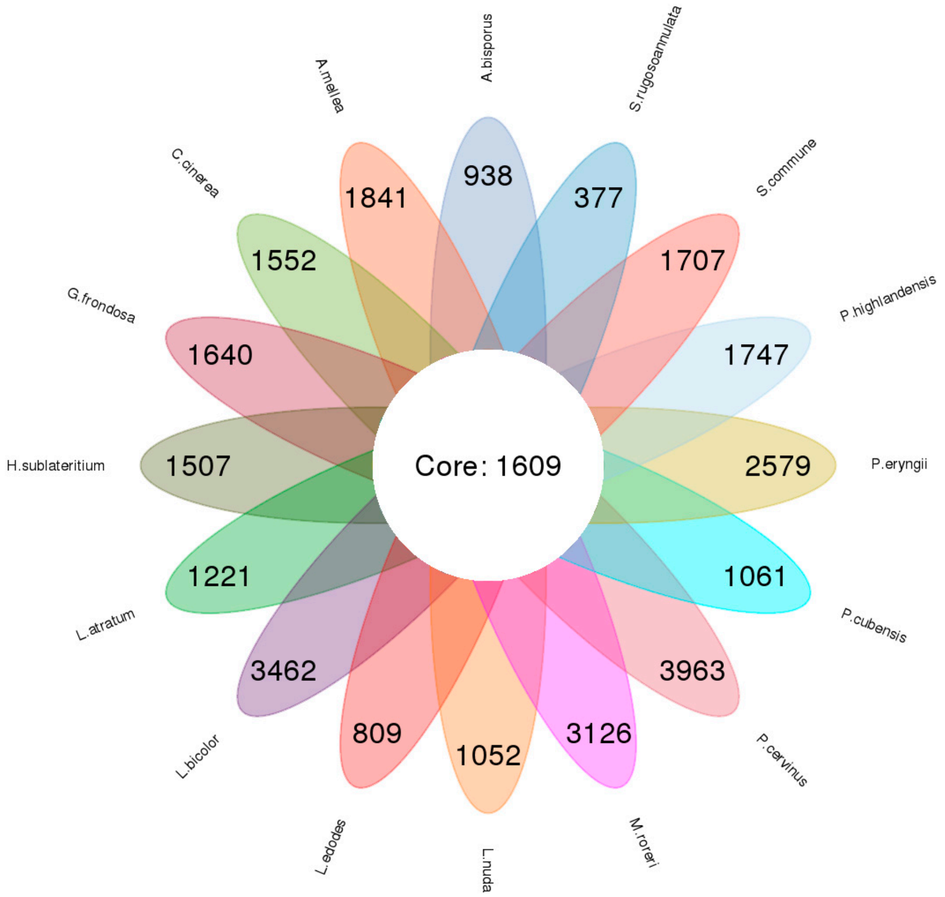

Figure S6. Venn diagram of orthologous gene families

Figure S7.

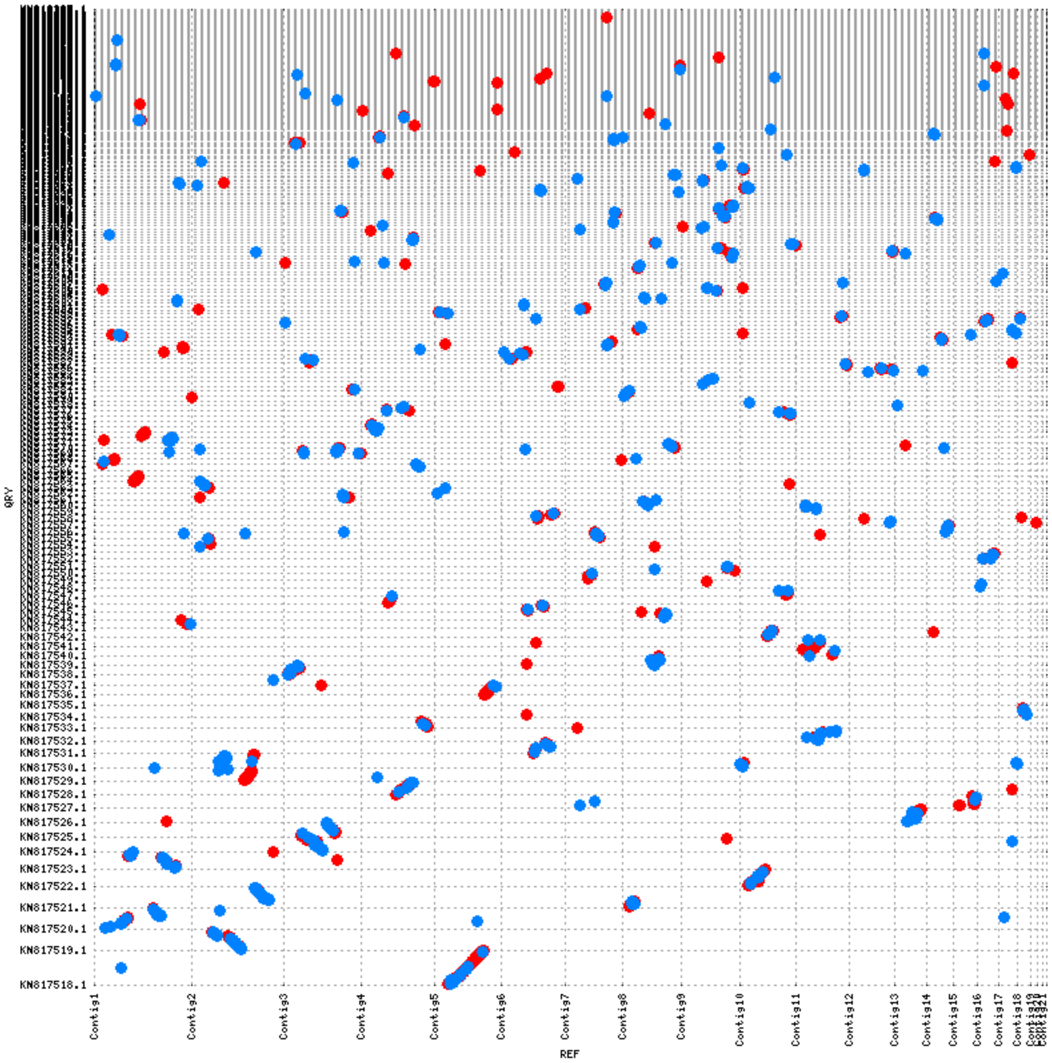

Figure S7. Colinear comparison of the whole genomes of *Stropharia rugosoannulata* and *Hypholoma sublateritium* using MUMmer plots

The red dots represent sequence collinearity, and the blue dots represent sequence inversion.

Figure S8.

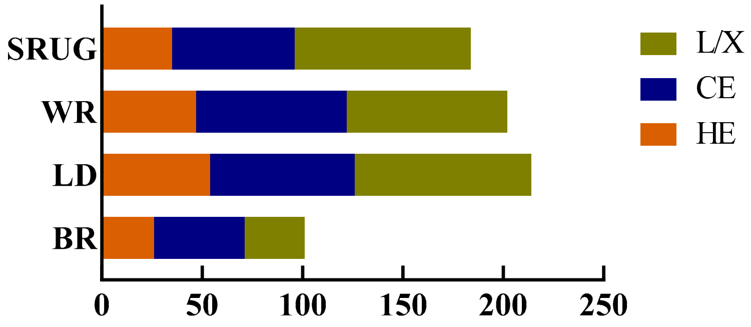

Figure S8. Comparison analysis of the number of predicted proteins involved in the degradation HE (hemicellulose), CE (cellulose), and L/X (lignin/xenobiotics)
The X-axis shows the number of genes and the Y-axis shows the nutritional strategies and *S. rugosoannulata*. LD: litter decomposers, BR: brown-rot fungi, WR: white-rot fungi, SRUG: *S. rugosoannulata*

Figure S9.

Cluster 1

Contig1, NRPS-like, Location: 4035697 - 4079731 nt (total 44035 nt)

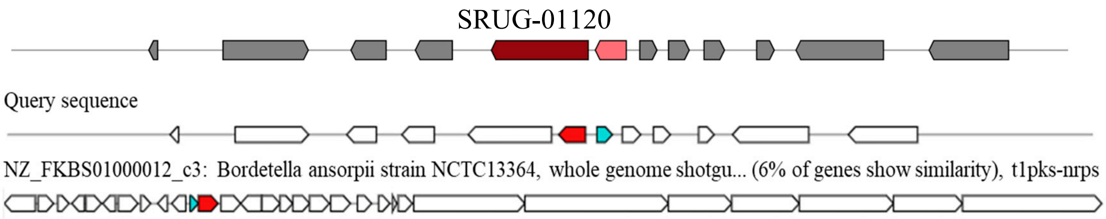

Cluster 2

Contig2, Region 2 -NRPS-like, Location: 338290 - 388136 nt (total 49847 nt)

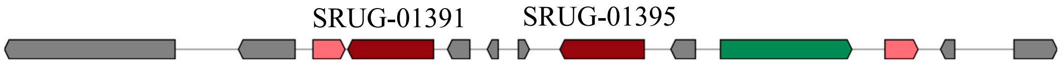

Cluster 3

Contig2, Region 2 - NRPS-like, Location: 408253 - 464478 nt (total 56226 nt)

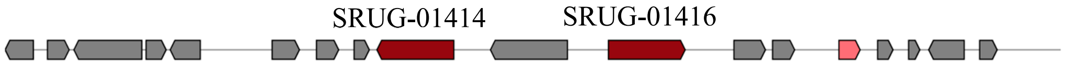

Cluster 4

Contig2, Region 3 - siderophore, Location 3397577 - 3409795 nt (total 12219 nt)

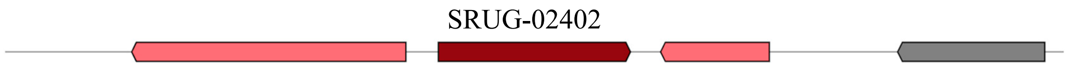

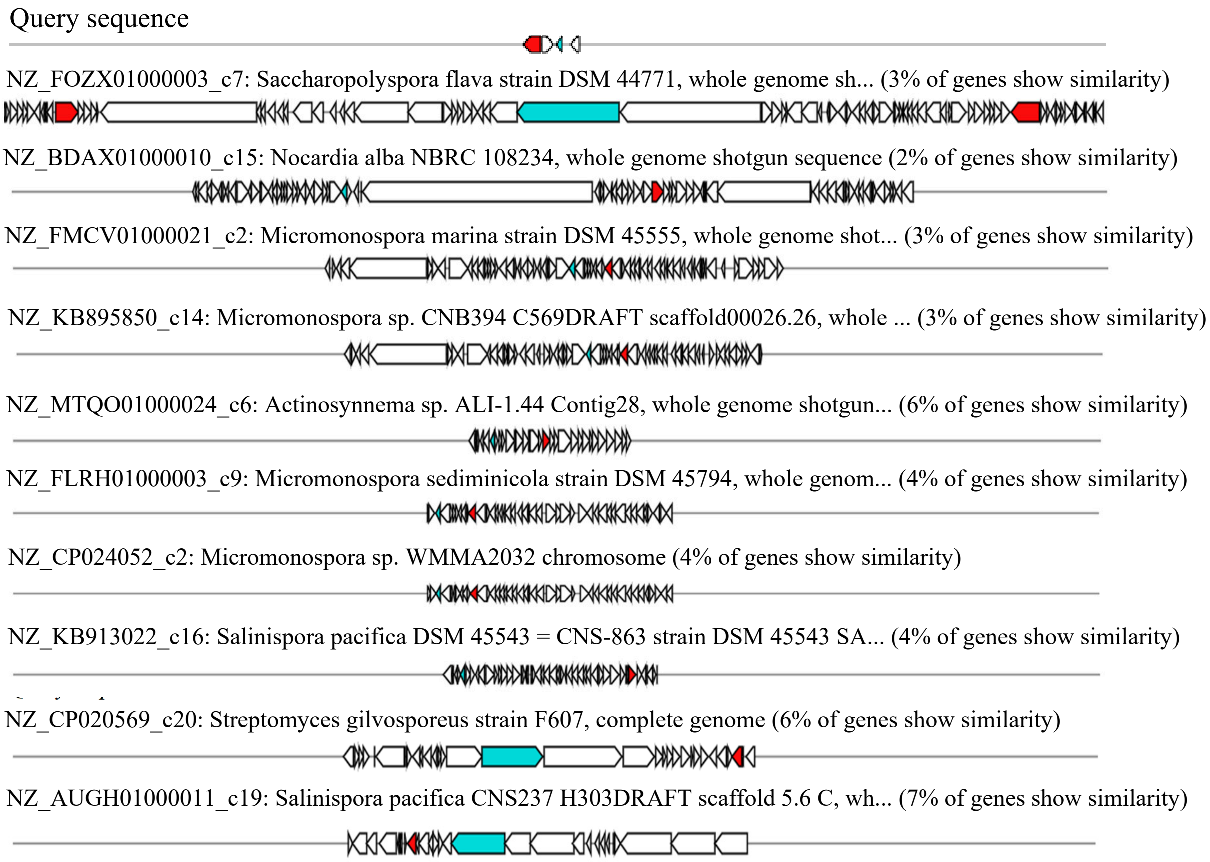

Cluster5

Contig3, Region 1 - terpene, Location: 666283 - 687725 nt (total 21443 nt)

SRUG-02831

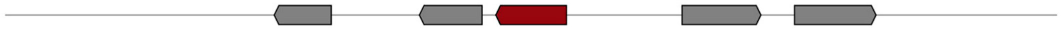

Cluster6

Contig3, Region 2 - NRPS, Location: 1783703 - 1831735 nt (total 48033 nt)

SRUG-03252

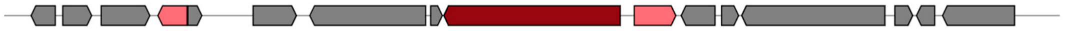

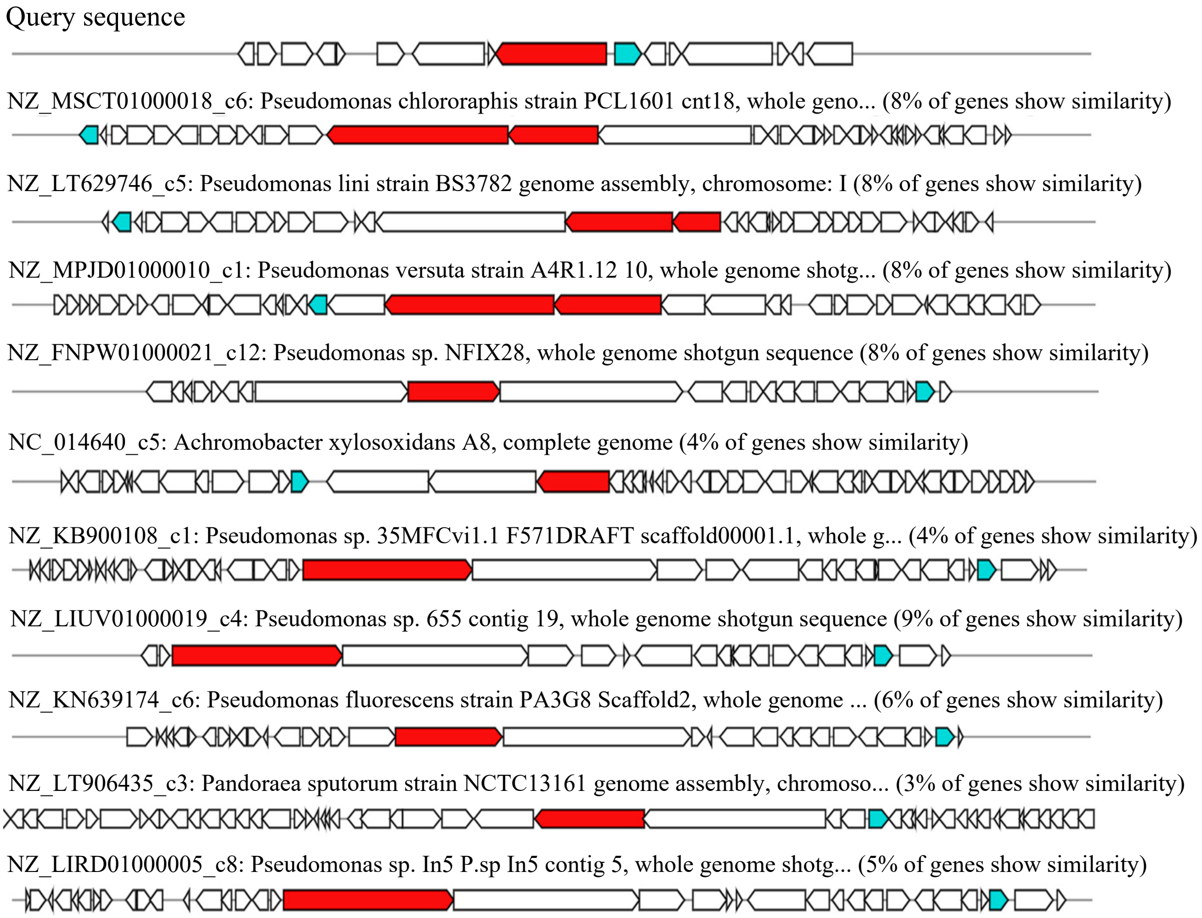

Cluster 7

Contig4, Region 1 – siderophore, Location: 1960289 - 1972449 nt (total: 12161 nt)

SRUG-04293

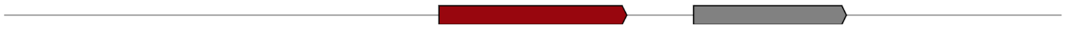

Cluster 8

Contig4, Region 2 – terpene, Location: 2395330 - 2416595 nt (total: 21266 nt)

SRUG-04425

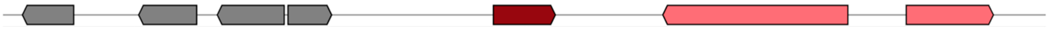

Cluster 9

Contig4 - Region 3 - NRPS-like, Location: 3572551 - 3616679 nt (total: 44129 nt)

SRUG-04722

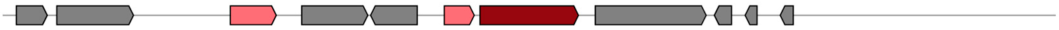

Cluster 10

Contig5, Region 1 – terpene, Location: 1053109 - 1074431 nt (total: 21323 nt)

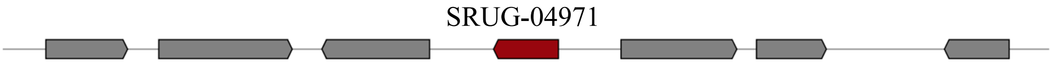

Cluster 11

Contig6, Region 1 – terpene, Location: 340380 - 355479 nt (total: 15100 nt)

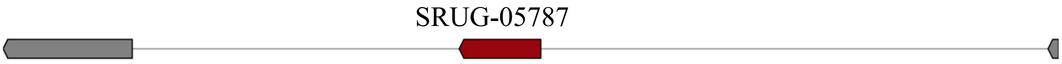

Cluster 12

Contig6, Region 2 – terpene, Location: 647505 - 679621 nt. (total: 32117 nt)

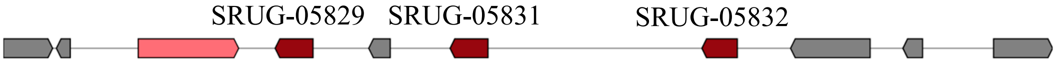

Cluster 13

Contig6, Region 3 – terpene, Location: 2077,902 - 2099118 nt (total: 21,217 nt)

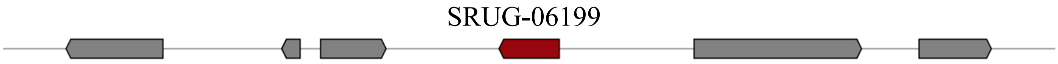

Cluster 14

Contig7, Region 1 – terpene, Location: 465221 - 485608 nt (total: 20388 nt)

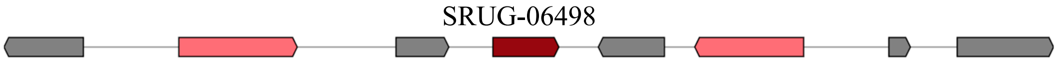

Cluster 15

Contig7, Region 2 - NRPS-like, Location: 1626863 - 1674587 nt (total: 47725 nt)

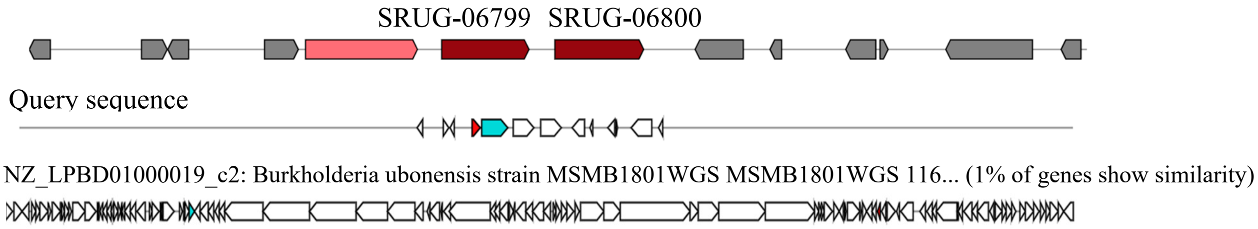

Cluster 16

Contig7, Region 3 - terpene, Location: 2536998 - 2555968 nt (total: 18971 nt)

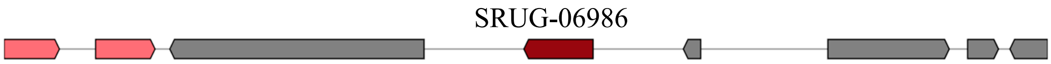

Cluster 17

Contig7, Region 4 – terpene, Location: 2567855 - 2589139 nt (total: 21285 nt)

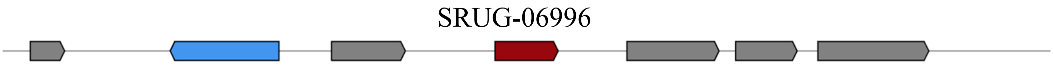

Cluster 18

Contig8, Region 1 - NRPS-like, Location: 239884 - 283810 nt (total: 43927 nt)

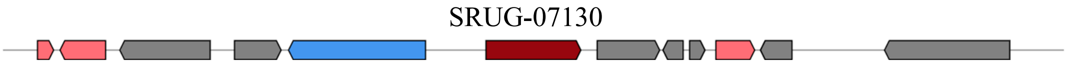

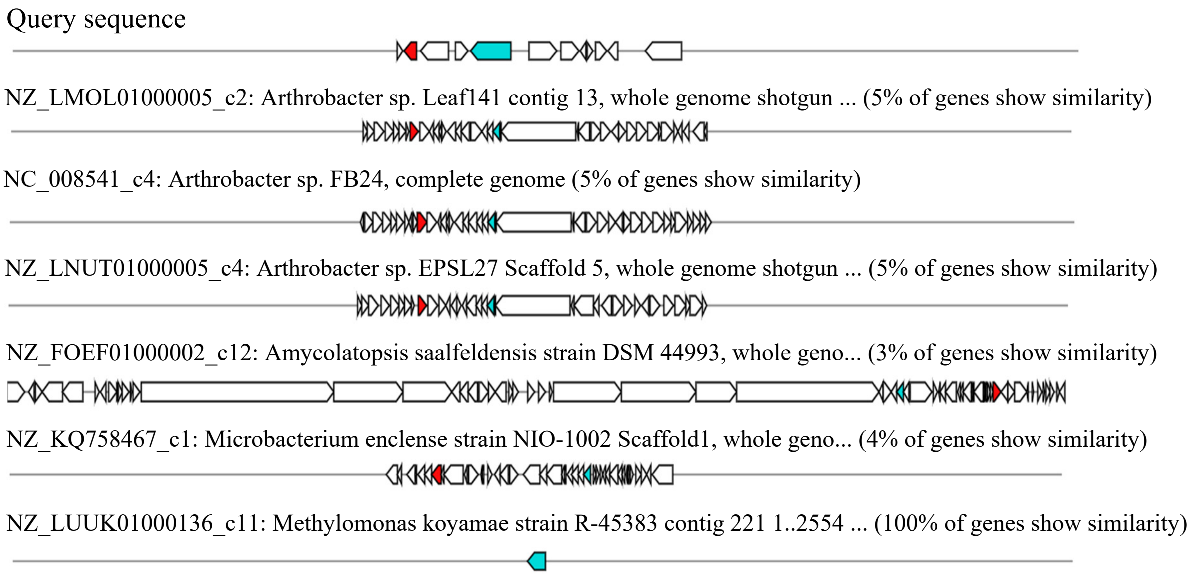

Cluster 19

Contig8, Region 2 – terpene, Location: 1148201 - 1164861 nt (total 16661 nt)

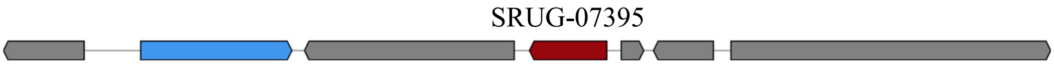

Cluster 20

Contig8, Region 3 – terpene, Location: 2224009 - 2245487 nt (total 21479 nt)

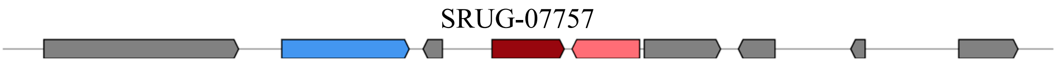

Cluster 21

Contig8, Region 4 - T1PKS, Location: 2256646 - 2303282 nt (total 46637 nt)

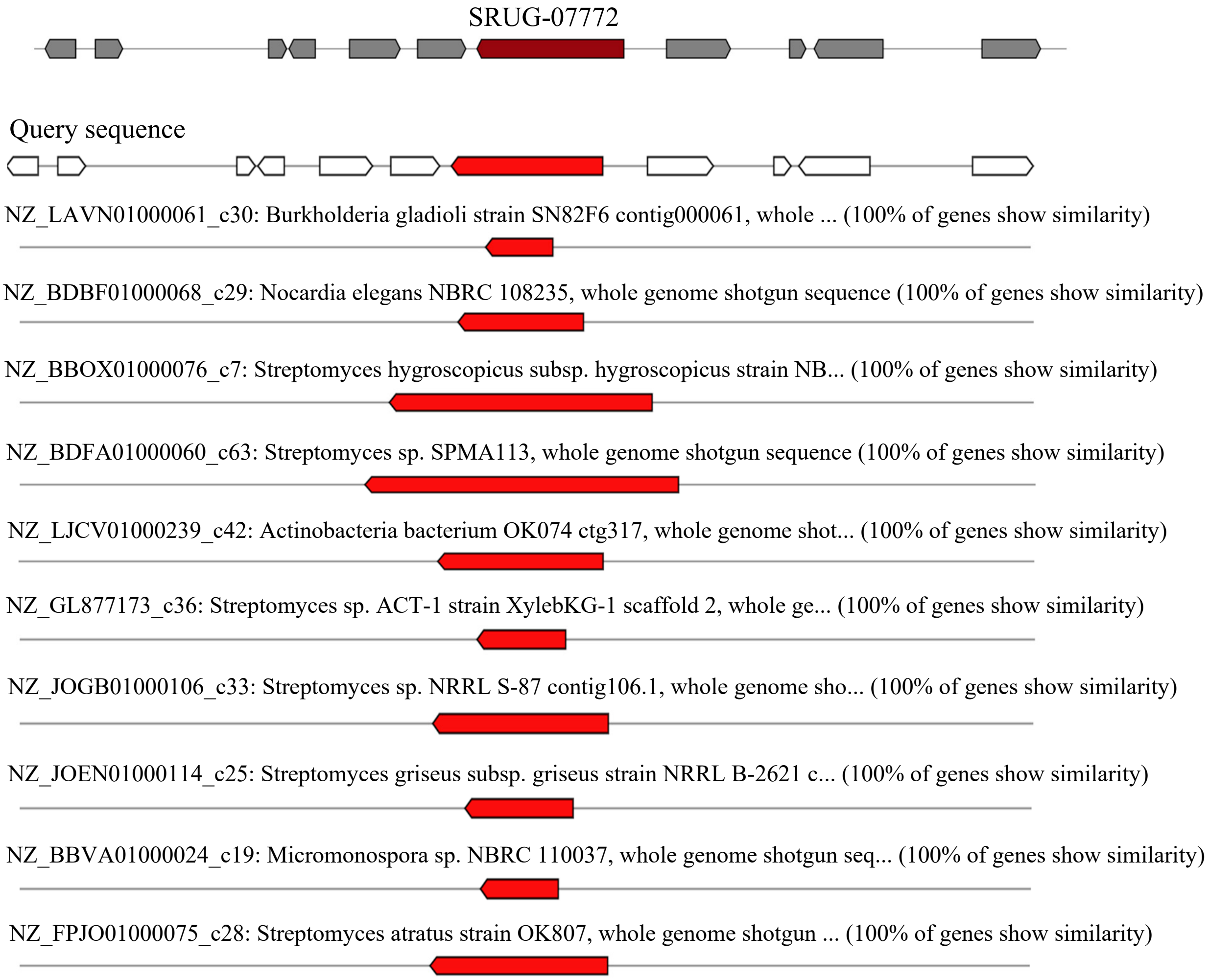

Cluster 22

Contig9, Region 1 - NRPS-like, Location: 233391 - 255448 nt (total: 22058 nt)

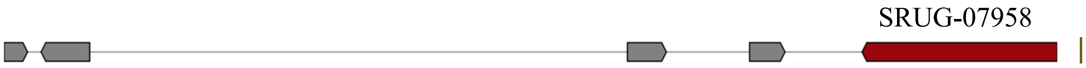

Cluster 23

Contig9, Region 2 – terpene, Location: 1580441 - 1600518 nt (total: 20078 nt)

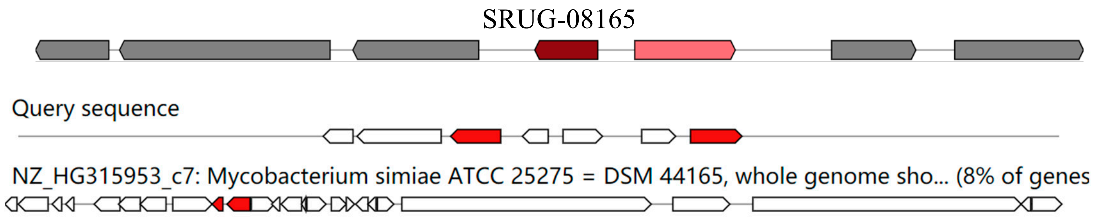

Cluster 24

Contig9, Region 3 – terpene, Location: 2160936 - 2182205 nt (total 21270 nt)

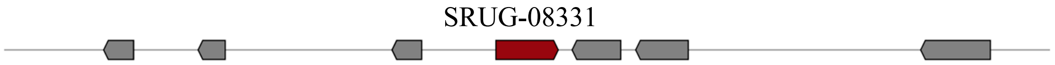

Cluster 25

Contig9, Region 4 - NRPS-like,T1PKS, Location: 2562731 - 2610976 nt (total: 48246 nt)

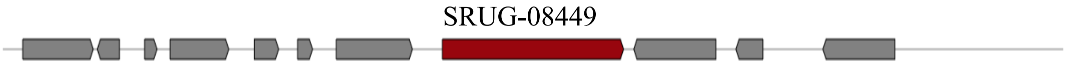

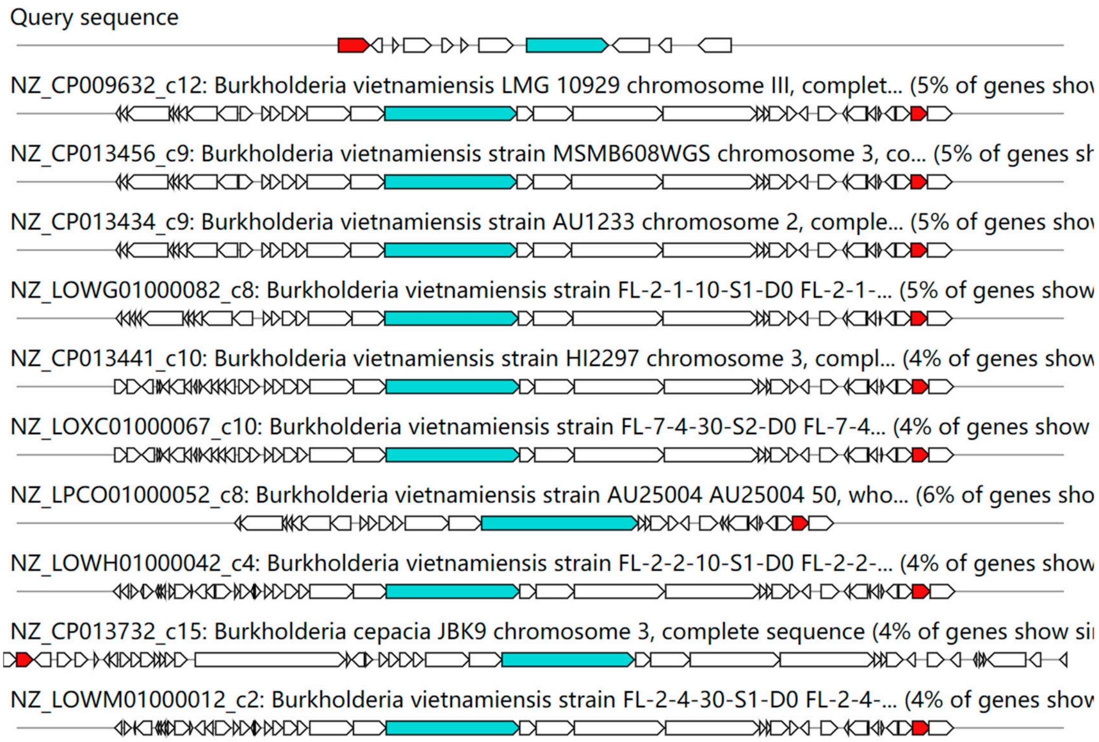

Cluster 26

Contig10, Region 1 - NRPS-like, Location: 2251608 - 2297150 nt (total 45543 nt)

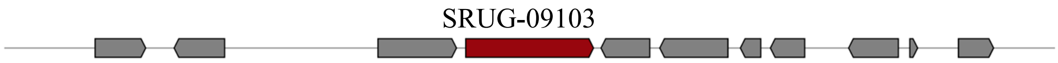

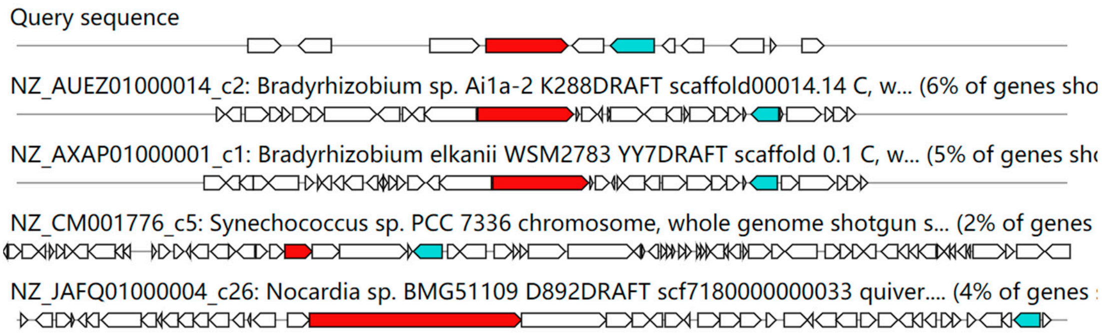

Cluster 27

Contig10, Region 2 – terpene, Location: 2384642 - 2406483 nt (total 21842 nt)

SRUG-09141

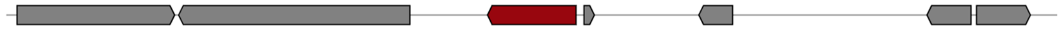

Cluster 28

Contig11, Region 1 - NRPS-like, Location: 1656079 - 1700094 nt (total 44016 nt)

SRUG-09635

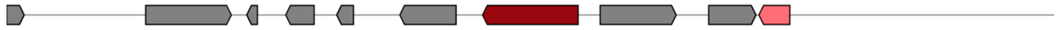

Cluster 29

Contig12, Region 1 – indole, Location: 679975 - 698519 nt (total 18545 nt)

SRUG-09987

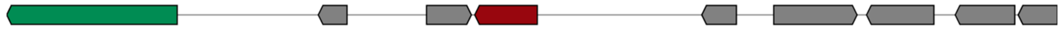

Cluster 30

Contig12, Region 2 – terpene, Location: 1482858 - 1503883 nt (total 21026 nt)

SRUG-10095

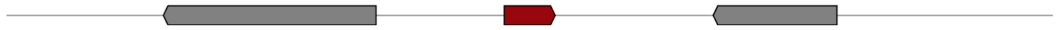

Cluster 31

Contig12, Region 3 – indole, Location: 1656043 - 1677263 nt (total 21221 nt)

SRUG-10137

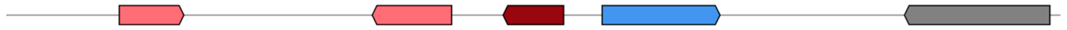

Cluster 32

Contig12, Region 4 - NRPS-like, Location: 2124080 - 2168664 nt (total 44585 nt)

SRUG-10224

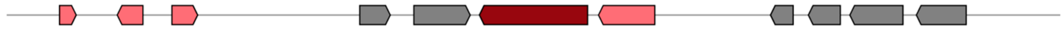

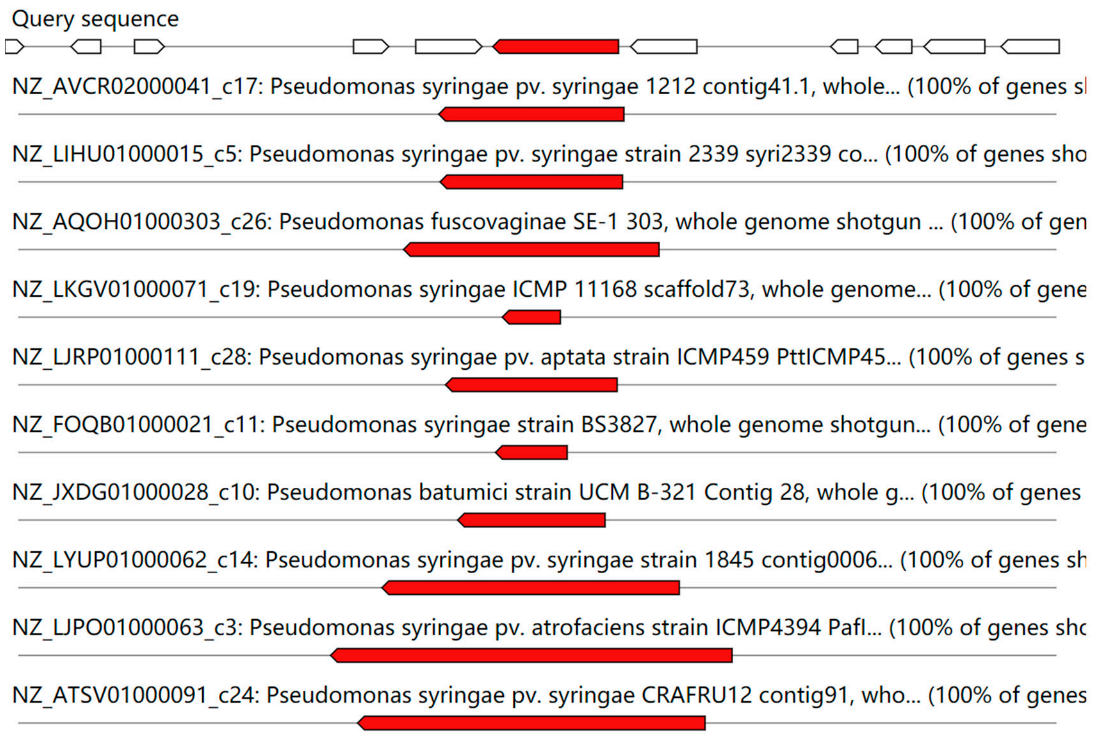

Cluster 33

Contig15,Region 1 – terpene, Location: 1153025 - 1174185 nt (total 21161 nt)

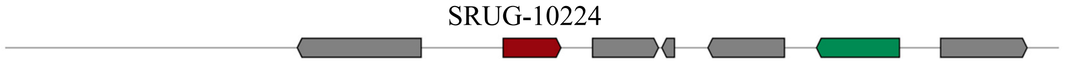

Cluster 34

Contig16, Region 1 – indole, Location: 513075 - 534267 nt. (total 21193 nt)

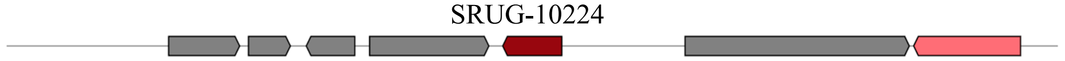

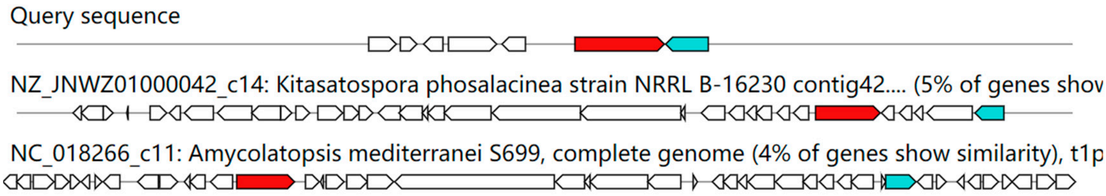

Figure S9. Biosynthetic gene clusters in the *Stropharia rugosoannulata* genome

The genes with predicted functions are: core biosynthetic genes ( ), additional biosynthetic genes ( ), transport-related genes ( ), regulatory genes ( ) and other genes ( ).

Table S1 Sequencing data used for the genome assembly

Library	Sequencing platform	Insert size	Raw data (Gb)	Clean data (GB)	Sequence coverage (X)	Application
Genome	Illumina HiSeq X Ten	350bp	7.06	6.52	129	Genome survey and assessment
Genome	PacBio SEQUEL	20 kb	14.57	14.55	266	Contig assembly
Transcriptome	Illumina HiSeq™	400bp	6.89	6.31	126	Genome annotation and assessment

Table S2 The genome information of comparative analysis species

Abbreviated taxon name	Classification		assembly size (Mbp)	Protein-coding genes	core genes	common genes	unique genes	Total repeat length (%)	GC content (%)
Agabi	Agaricaceae (Agaricales, Basidiomycota)	Agaricoid	30.74	11278	1609	8731	938	13.83	46.5
Armmel	Physalacriaceae (Agaricales, Basidiomycota)	Marasmioid	70.86	15553	1609	12103	1841	10.45	48
Copci	Psathyrellaceae (Agaricales, Basidiomycota)	Agaricoid	36.19	13356	1609	10195	1552	6.678	51.6
Grifr	Grifolaceae (Polyporales, Basidiomycota)	Polyporales	40.58	15039	1609	11790	1640	18.08	49.5
Hypsu	Strophariaceae (Agaricales, Basidiomycota)	Agaricoid	48	17771	1609	14655	1507	1.1	51
Lacbi	Hydnangiaceae (Agaricales, Basidiomycota)	Agaricoid	64.87	18215	1609	13144	3462	22.05	47.1
Lened	Omphalotaceae (Agaricales, Basidiomycota)	Marasmioid	46.1	10927	1609	8509	809	17.7	45.33
Lepnud	Tricholomataceae (Agaricales, Basidiomycota)	Tricholomatoid	43.49	14876	1609	12215	1052	8.115	46.2
Lyoat	Lyophyllaceae (Agaricales, Basidiomycota)	Tricholomatoid	64.89	15046	1609	12216	1221	6.61	51.3
Monro	Marasmiaceae (Agaricales, Basidiomycota)	Marasmioid	54.79	20131	1609	15396	3126	13.13	46.7
Phohig	Strophariaceae (Agaricales, Basidiomycota)	Agaricoid	66.04	17628	1609	14272	1747	5.51	49
Pleery	Pleurotaceae (Agaricales, Basidiomycota)	Pluteoid	47.26	15954	1609	11766	2579	18.187	49.35

Plucer	Pluteaceae (Agaricales, Basidiomycota)	Pluteoid	52.19	19812	1609	14240	3963	19.4	48.7
Psicub	Hymenogastraceae (Agaricales, Basidiomycota)	Agaricoid	41.3	13236	1609	10566	1061	14.2	46
Schco	Schizophyllaceae (Agaricales, Basidiomycota)	Marasmioid	38.15	13193	1609	9877	1707	11.2	57.529
Strru	Strophariaceae (Agaricales, Basidiomycota)	Agaricoid	48.33	11750	1609	9764	377	19.91	47.35

Abbreviated taxon name: Agabi: *Agaricus bisporus*; Armmel: *Armillaria mellea*; Copci: *Coprinopsis cinerea*; Grifr: *Grifola frondosa*; Hypsu: *Hypholoma sublateritium*; Lacbi: *Laccaria bicolor*; Lened: *Lentinula edodes*; Lepnud: *Lepista nuda*; Lyoat: *Lyophyllum atratum*; Monro: *Moniliophthora roreri*; Phohig: *Pholiota highlandensis*; Pleery: *Pleurotus eryngii*; Plucer: *Pluteus cervinus*; Psicub: *Psilocybe cubensis*; Schco: *Schizophyllum commune*; Strru: *Stropharia rugosoannulata*.

Table S3 The assembled results of *Stropharia rugosoannulata* genome

#	Stat Type	Contig Length	Contig Number	Scaffold Length	Scaffold Number
	N50	2961130	7	2961130	7
	N60	2955062	8	2955062	8
	N70	2902069	10	2902069	10
	N80	2310885	12	2310885	12
	N90	1348662	14	1348662	14
	Longest	4928370	1	4928370	1
	Total	48331048	21	48331048	21
	Length>=1kb	48331048	21	48331048	21
	Length>=2kb	48331048	21	48331048	21
	Length>=5kb	48331048	21	48331048	21

Table S4 Summary statistics for telomere repeats in the assembly.

Contig	Contig length	Telomere length	Number of Telomeric Repeats	Strand	Start	end
Contig 1	4928370	91	13	CCCTAAA	3	93
Contig 2	4665326	121	17	CCCTAAA	9	129
Contig 3	3966484	105	15	CCCTAAA	5	109
		175	25	TTTAGGG	3966308	3966482
Contig 4	3692652	140	20	TTTAGGG	3692511	3692650
Contig 5	3360661	/	/		/	/
Contig 6	3254096	154	22	TTTAGGG	3253937	3254090
Contig 7	2961130	104	14	CCCTAAA	1	104
		119	17	TTTAGGG	2961008	2961126
Contig 8	2955062	182	26	TTTAGGG	2954879	2955061
Contig 9	2950788	133	19	CCCTAAA	5	137
Contig 10	2902069	175	25	TTTAGGG	2901895	2902069
Contig 11	2694211	91	13	CCCTAAA	3	93
Contig 12	2310885	56	8	CCCTAAA	6	61
Contig 13	1579960	/	/		/	/
Contig 14	1348662	119	17	CCCTAAA	1	119
Contig 15	1265009	/	/		/	/
Contig 16	1082040	126	18	CCCTAAA	1	126
Contig 17	950328	/	/		/	/
Contig 18	647927	97	13	TTTAGGG	647828	647924
Contig 19	343027	147	21	TTTAGGG	342880	343026
Contig 20	289444	/	/		/	/
Contig 21	182917	126	18	CCCTAAA	3	128

Table S5 Genome information of SR68 and MG69

**Characteristics**	**SR-68**	**MG69**
Genome assembly size (Mb)	48.33	49.5
Sequencing depth	266	51
Contigs	21	17725
Longest contig (kb)	4928	131
N50 (kb)	2961	8.32
GC (%)	47.35	47.8
Protein-coding genes	11750	17683
Busco result	94.14%	87.60%
Sequencing method	PacBio, Illumina	Illumina

Table S6-1 Prediction results of gene structure

Gene set	Total number of genes	Average gene length(bp)	Average CDS length(bp)	Average exons number per gene	Average exon length(bp)	Average intron length(bp)
De novo	12673	1934.13	1419.55	7.02	202.12	85.43
Homology	11747	2647.74	1435.65	6	239.34	242.5
RNA-seq	6376	2010.14	1647.83	6.79	242.79	62.61
Final set	11750	1942.05	1455.07	6.25	232.84	92.77

Table S6-2 BUSCO prediction

Type	Number	Percent (%)
Complete BUSCOs (C)	273	94.14
Complete and single-copy BUSCOs (S)	271	93.45
Complete and duplicated BUSCOs (D)	2	0.69
Fragmented BUSCOs (F)	4	1.38
Missing BUSCOs (M)	13	4.48
Total BUSCO groups searched	290	100

Table S6-3 Gene function annotation

Type	Database	Number	Percent (%)
Annotation	Swissprot	6746	57.41
	KEGG	3826	32.56
	KOG	3280	27.91
	GO	5496	46.77
	NR	11359	96.67

Total	Annotated	11377		96.83
	Gene	11750	-	

Table S7 GO analysis of secreted proteins

Ontology	GO ID	GO Term	P value	FDR	Gene Ratio	BgRatio
MF	GO:0016787	hydrolase activity	9.58E-48	1.81E-45	207	945
CC	GO:0005576	extracellular region	4.03E-47	1.29E-45	41	47
CC	GO:0005618	cell wall	8.42E-45	8.98E-44	41	50
CC	GO:0030312	external encapsulating structure	8.42E-45	8.98E-44	41	50
CC	GO:0009277	fungal-type cell wall	3.86E-42	3.09E-41	39	48
BP	GO:0005975	carbohydrate metabolic process	5.45E-41	1.21E-38	97	259
MF	GO:0004553	hydrolase activity, hydrolyzing O-glycosyl compounds	1.17E-38	1.10E-36	72	149
MF	GO:0016798	hydrolase activity, acting on glycosyl bonds	1.78E-38	1.12E-36	73	154
MF	GO:0001871	pattern binding	8.49E-37	3.21E-35	36	38
MF	GO:0030247	polysaccharide binding	8.49E-37	3.21E-35	36	38
MF	GO:0003824	catalytic activity	2.58E-33	7.12E-32	346	2710
MF	GO:0005199	structural constituent of cell wall	2.64E-33	7.12E-32	39	49
CC	GO:0071944	cell periphery	6.94E-33	4.44E-32	41	76
MF	GO:0030246	carbohydrate binding	2.20E-32	5.20E-31	45	68
MF	GO:0030248	cellulose binding	1.78E-30	3.73E-29	29	30
MF	GO:0070011	peptidase activity, acting on L-amino acid peptides	1.15E-29	2.16E-28	74	203
MF	GO:0008233	peptidase activity	4.46E-29	7.67E-28	75	212
MF	GO:0008236	serine-type peptidase activity	1.08E-23	1.57E-22	37	64
MF	GO:0017171	serine hydrolase activity	1.08E-23	1.57E-22	37	64
BP	GO:0008152	metabolic process	2.73E-23	3.03E-21	283	2483
MF	GO:0004175	endopeptidase activity	7.30E-22	9.86E-21	46	108
MF	GO:0004601	peroxidase activity	1.96E-21	2.47E-20	32	53
MF	GO:0016684	oxidoreductase activity, acting on peroxide as acceptor	9.08E-20	1.07E-18	32	58
BP	GO:0006508	proteolysis	2.61E-19	1.93E-17	59	191

MF	GO:0016209	antioxidant activity	1.49E-17	1.66E-16	32	66
MF	GO:0004180	carboxypeptidase activity	1.22E-15	1.28E-14	18	23
MF	GO:0004185	serine-type carboxypeptidase activity	2.82E-15	2.80E-14	17	21
MF	GO:0004190	aspartic-type endopeptidase activity	1.50E-14	1.35E-13	19	28
MF	GO:0070001	aspartic-type peptidase activity	1.50E-14	1.35E-13	19	28
BP	GO:0006979	response to oxidative stress	3.24E-14	1.80E-12	23	42
MF	GO:0070008	serine-type exopeptidase activity	4.04E-14	3.47E-13	17	23
MF	GO:0050660	flavin adenine dinucleotide binding	3.97E-12	3.27E-11	40	141
MF	GO:0008238	exopeptidase activity	6.07E-10	4.78E-09	18	39
MF	GO:0005198	structural molecule activity	4.22E-09	3.19E-08	39	167
CC	GO:0005615	extracellular space	7.88E-09	3.60E-08	8	10
CC	GO:0044421	extracellular region part	7.88E-09	3.60E-08	8	10
BP	GO:0000272	polysaccharide catabolic process	4.32E-08	1.92E-06	7	7
MF	GO:0016614	oxidoreductase activity, acting on CH-OH group of donors	6.31E-08	4.58E-07	24	83
BP	GO:0055114	oxidation-reduction process	7.81E-08	2.89E-06	95	652
MF	GO:0016491	oxidoreductase activity	9.72E-08	6.80E-07	105	754
MF	GO:0008237	metallopeptidase activity	1.43E-07	9.50E-07	18	52
MF	GO:0004252	serine-type endopeptidase activity	1.46E-07	9.50E-07	13	28
MF	GO:0004222	metalloendopeptidase activity	1.93E-07	1.22E-06	14	33
MF	GO:0004650	polygalacturonase activity	2.60E-07	1.54E-06	7	8
MF	GO:2001070	starch binding	2.60E-07	1.54E-06	7	8
MF	GO:0020037	heme binding	1.87E-06	1.04E-05	39	207
MF	GO:0046906	tetrapyrrole binding	1.87E-06	1.04E-05	39	207
MF	GO:0004339	glucan 1,4-alpha-glucosidase activity	4.79E-06	2.59E-05	5	5
MF	GO:0016788	hydrolase activity, acting on ester bonds	4.96E-06	2.61E-05	34	176
BP	GO:0005976	polysaccharide metabolic process	1.04E-05	0.00032875	7	11

MF	GO:0015926	glucosidase activity	2.67E-05	0.00013642	5	6
MF	GO:0050662	coenzyme binding	2.76E-05	0.00013751	40	239
BP	GO:0071554	cell wall organization or biogenesis	3.27E-05	0.00090622	6	9
MF	GO:0071949	FAD binding	0.000103	0.0004991	15	59
MF	GO:0052689	carboxylic ester hydrolase activity	0.000221	0.00104336	8	21
BP	GO:0006950	response to stress	0.000246	0.00605579	25	134
BP	GO:0016052	carbohydrate catabolic process	0.000393	0.00872251	8	22
MF	GO:0003796	lysozyme activity	0.000648	0.00291392	3	3
MF	GO:0045735	nutrient reservoir activity	0.000648	0.00291392	3	3
MF	GO:0005507	copper ion binding	0.000702	0.00308755	7	19
BP	GO:0000270	peptidoglycan metabolic process	0.000711	0.00876893	3	3
BP	GO:0006026	aminoglycan catabolic process	0.000711	0.00876893	3	3
BP	GO:0006027	glycosaminoglycan catabolic process	0.000711	0.00876893	3	3
BP	GO:0009253	peptidoglycan catabolic process	0.000711	0.00876893	3	3
BP	GO:0016998	cell wall macromolecule catabolic process	0.000711	0.00876893	3	3
BP	GO:0030203	glycosaminoglycan metabolic process	0.000711	0.00876893	3	3
BP	GO:0033609	oxalate metabolic process	0.000711	0.00876893	3	3
BP	GO:0044036	cell wall macromolecule metabolic process	0.000711	0.00876893	3	3
MF	GO:0008199	ferric iron binding	0.000726	0.00311721	4	6
MF	GO:0048037	cofactor binding	0.001034	0.00434197	41	291
BP	GO:0044238	primary metabolic process	0.001064	0.01243725	182	1748
BP	GO:0071704	organic substance metabolic process	0.001727	0.01917044	190	1853
MF	GO:0016702	oxidoreductase activity, acting on single donors with incorporation of molecular oxygen, incorporation of two atoms of oxygen	0.002267	0.00931439	5	12
MF	GO:0030599	pectinesterase activity	0.002423	0.00974283	3	4
BP	GO:0042545	cell wall modification	0.002655	0.02562442	3	4

BP	GO:0045229	external encapsulating structure organization	0.002655	0.02562442	3	4
BP	GO:0071555	cell wall organization	0.002655	0.02562442	3	4
CC	GO:0042597	periplasmic space	0.003972	0.01588615	2	2
MF	GO:0051213	dioxygenase activity	0.004953	0.01950208	5	14
MF	GO:0016892	endoribonuclease activity, producing 3'-phosphomonoesters	0.005667	0.02186034	3	5
MF	GO:0016810	hydrolase activity, acting on carbon-nitrogen (but not peptide) bonds	0.006952	0.02444342	12	63
MF	GO:0003756	protein disulfide isomerase activity	0.007501	0.02444342	2	2
MF	GO:0004104	cholinesterase activity	0.007501	0.02444342	2	2
MF	GO:0004560	alpha-L-fucosidase activity	0.007501	0.02444342	2	2
MF	GO:0004767	sphingomyelin phosphodiesterase activity	0.007501	0.02444342	2	2
MF	GO:0015928	fucosidase activity	0.007501	0.02444342	2	2
MF	GO:0016864	intramolecular oxidoreductase activity, transposing S-S bonds	0.007501	0.02444342	2	2
MF	GO:0033897	ribonuclease T2 activity	0.007501	0.02444342	2	2
MF	GO:0050525	cutinase activity	0.007501	0.02444342	2	2
MF	GO:0008081	phosphoric diester hydrolase activity	0.009205	0.02948731	6	22
MF	GO:0008061	chitin binding	0.011231	0.03537741	4	11

Table S8 Number of genes in a gene family

Species name	Genes Number	Genes number in families	Unclustered genes number	Family number	Unique Families number	Average genes number per family
Agaricus bisporus	10,331	9,018	1,313	6,698	165	1.35
Armillaria mellea	15,553	13,129	2,424	8,495	516	1.55
Coprinopsis cinerea	13,350	10,662	2,688	7,763	417	1.37
Grifola frondosa	15,038	10,172	4,866	7,157	472	1.42
Hypholoma sublateritium	17,569	13,251	4,318	9,343	477	1.42
Lyophyllum atratum	14,959	12,990	1,969	8,777	412	1.48
Laccaria bicolor	18,215	14,069	4,146	8,342	944	1.69
Lentinula edodes	13,998	9,588	4,410	7,545	267	1.27
Lepista nuda	14,708	12,056	2,652	8,749	331	1.38
Moniliophthora roreri	19,855	15,660	4,195	8,287	686	1.89
Pluteus cervinus	19,497	15,685	3,812	8,508	701	1.84
Psilocybe cubensis	13,236	11,438	1,798	8,421	303	1.36
Pleurotus eryngii	15,758	12,548	3,210	8,276	646	1.52
Pholiota highlandensis	17,628	14,910	2,718	8,997	564	1.66
Schizophyllum commune	13,235	11,230	2,005	7,699	426	1.46
Stropharia rugosoannulata	11,750	10,967	783	8,341	131	1.31

Table S9 Putative peroxidase-encoding genes of the 63 Agricomycotina[35]

Nutritional strategies	Reference Name	Subphylum	Haem peroxidase																Non-haem peroxidase									
			Haloperoxidase (haem)	Linoleate diol synthase (PGHS like)	Catalase	Cytochrome C peroxidase	DyP-type peroxidase D	Hybrid Ascorbate-Cytochrome C peroxidase	Lignin peroxidase	Manganese peroxidase	Versatile peroxidase	Other class II peroxidase	NoxA	NoxB	NoxC	Doux	Rbohs	total	Atypical 2-Cysteine peroxiredoxin (typeII, typeV)	Atypical 2-Cysteine peroxiredoxin (typeQ, BCP)	1-Cysteine peroxiredoxin (1CysPrx)	Carboxymuconolactone decarboxylase	Fungi-Bacteria glutathione peroxidase	Typical 2-Cysteine peroxiredoxin	No haem, Vanadium chloroperoxidase	NoxR		total
biotroph (ectomycorrhizal)	Amamu	Agricomycotina	4	3	2	1	2	1	0	0	0	0	1	1	0	0	0	15	2	2	1	1	1	2	0	1	9	25
biotroph (ectomycorrhizal)	Hebcy	Agricomycotina	7	3	1	1	2	4	0	3	0	0	1	1	0	0	0	23	2	2	1	1	1	1	0	1	8	32
biotroph (ectomycorrhizal)	Lacam	Agricomycotina	8	2	1	1	2	1	0	1	0	0	1	1	0	0	0	18	2	2	1	1	2	1	0	1	9	28
biotroph (ectomycorrhizal)	Lacbi	Agricomycotina	5	2	1	1	2	1	0	1	0	0	1	1	0	0	0	15	2	2	1	1	1	1	0	1	8	24
biotroph (ectomycorrhizal)	Paxin	Agricomycotina	4	2	4	2	0	0	0	0	0	0	1	1	0	0	0	14	4	2	1	0	1	2	0	1	10	25
biotroph (ectomycorrhizal)	Paxru	Agricomycotina	4	2	2	1	0	0	0	0	0	0	1	1	0	0	0	11	2	1	1	0	1	1	0	1	6	18
biotroph (ectomycorrhizal)	Pilcr	Agricomycotina	11	4	2	1	4	0	0	0	0	1	1	1	0	0	0	25	1	3	1	3	1	1	0	3	10	38
biotroph (ectomycorrhizal)	Pismi	Agricomycotina	5	1	1	1	0	0	0	0	0	0	1	1	0	0	0	10	3	2	1	0	1	1	0	1	8	19
biotroph (ectomycorrhizal)	Pisti	Agricomycotina	4	1	1	1	1	0	0	0	0	0	1	1	0	0	0	10	3	2	1	0	1	1	0	1	8	19
biotroph (root endophyte)	Pirin	Agricomycotina	2	2	1	1	2	0	0	0	0	0	1	1	0	0	0	10	1	1	1	0	1		0	1	4	15

biotroph (ectomycorrhizal)	Sclci	Agricomycotina	4	1	1	1	1	0	0	0	0	0	1	1	0	0	0	10	2	2	3	0	1	1	0	1	9	20
biotroph (ectomycorrhizal)	Suibr	Agricomycotina	5	2	3	1	1	0	0	0	0	0	1	1	0	0	0	14	2	2	1	0	1	2	0	1	8	23
biotroph (ectomycorrhizal)	Suilu	Agricomycotina	5	2	3	1	1	0	0	0	0	0	1	1	0	0	0	14	2	2	1	0	1	1	0	1	7	22
biotroph (orchid symbiont)	Sebve	Agricomycotina	0	3	1	1	3	0	0	0	0	0	1	1	0	0	0	10	1	1	1	0	1	0	0	1	4	15
biotroph (mycoparasite)	Treme	Agricomycotina	0	0	1	2	0	0	0	0	0	0	1	1	0	0	0	5	1	2	1	0	2	1	0	1	7	13
biotroph (orchid symbiont)	Tulca	Agricomycotina	2	4	2	1	2	0	0	0	0	0	1	2	0	0	0	14	4	3	1	0	1	1	0	3	10	27
BR	Calco	Agricomycotina	7	3	2	1	0	0	0	0	0	0	1	1	0	0	0	15	2	3	1	0		1	0	1	7	23
BR	Calvi	Agricomycotina	6	2	3	1		0	0	0	0	0	1	1	0	0	0	14	1	2	1	0	1	1	0	1	6	21
BR	Conpu	Agricomycotina	2	2	3	1	0	0	0	0	0	0	1	1	0	0	0	10	2	2	1	3	1	1	0	0	10	20
BR	Dacsp	Agricomycotina	6	3	3	1	0	0	0	0	0	0	1	1	0	0	0	15	1	3	1	0	1	1	0	1	7	23
BR	Daequ	Agricomycotina	4	2	4	1	0	0	0	0	0	1	1	1	0	0	0	14	2	2	1	0	1	1	0	1	7	22
BR	Fishe	Agricomycotina	3	2	2	1	0	0	0	0	0	0	0	1	0	0	0	9	2	2	1	1	1	1	0	1	8	18
BR	Fompi	Agricomycotina	4	5	3	1	0	0	0	0	0	1	1	1	0	0	0	16	2	2	1	0	1	1	0	1	7	24
BR	Glotr	Agricomycotina	6	3	4	1	0	0	0	0	0	0	1	1	0	0	0	16	2	2	1	0	1	1	0	1	7	24
BR	Hydpi	Agricomycotina	4	2	3	1	1	0	0	0	0	0	0	1	0	0	0	12	2	2	1	0	1	1	0	1	7	20
BR	Laesu	Agricomycotina	5	6	4	1	0	0	0	0	0	1	1	1	0	0	0	19	2	2	1	0	1	1	0	1	7	27
BR	Neole	Agricomycotina	9	3	3	1	0	0	0	0	0	0	1	1	0	0	0	18	2	2	1	0	1	1	0	1	7	26
BR	Pospl	Agricomycotina	8	4	8	1	2	0	0	0	0	2	2	2	0	0	0	29	4	1	2	0	2	2	0	2	11	42
BR	Serla	Agricomycotina	3	2	4	1	0	0	0	0	0	0	1	1	0	0	0	12	2	2	1	0	1	1	0	1	7	20
BR	Wolco	Agricomycotina	5	2	4	1	0	0	0	0	0	1	1	1	0	0	0	15	2	2	1	0	1	1	0	1	7	23
LD	Agabi	Agricomycotina	24	2	3	1	0	2	0	2	0	0	1	1	0	0	0	36	2	2	1	1	1	1	0	1	8	45
LD	Amath	Agricomycotina	4	4	1	1	1	1	0	0	0	0	1	1	0	0	0	14	3	2	1	1	1	1	0	1	9	24
LD	Copci	Agricomycotina	8	2	4	1	3	2	0	0	0	1	1	1	0	0	0	23	2	2	1	1	1	1	0	1	8	32
LD	Gymlu	Agricomycotina	14	2	5	1	12	8	0	5	0	0	1	2	0	0	0	50	2	2	5	2	1	2	0	1	14	65
WR	Aursu	Agricomycotina	16	2	3	1	11	4	0	18	0	0	1	1	0	0	0	57	2	2	1	2	1	1	0	2	9	68

WR	Bjead	Agricomycotina	4	3	4	1	12	0	12	7	1		1	1	0	0	0	46	2	2	2	1	1	1	0	1	9	56
WR	Botbo	Agricomycotina	7	4	3	1	3	0	0	0	0	0	1	1	0	0	0	20	3	1	1	0	1	1	0	1	7	28
WR	Cylto	Agricomycotina	5	2	3	1	0	1	0	0	0	0	1	1	0	0	0	14	2	1	1	1	1	2	0	1	8	23
WR	Dicsq	Agricomycotina	4	3	3	1	1	1	0	9	3	0	1	1	0	0	0	27	3	3	1	4	1	1	0	1	13	41
WR	Exigl	Agricomycotina	30	2	3	1	0	11	0	34	0	0	1	1	0	0	0	83	9	3	1	1	1	1	0	2	16	101
WR	Fibsp	Agricomycotina	35	5	5	1	2	0	0	0	0	0	2	1	0	0	0	51	4	4	1	1	1	1	0	2	12	65
WR	Fomme	Agricomycotina	4	3	2	1	3	0	0	17	0	0	1	1	0	0	0	32	2	2	1	1	1	1	0	1	8	41
WR	Galma	Agricomycotina	24	7	4	1	5	10	0	23	0	0	1	2	0	0	0	77	2	2	2	1	2	1	0	1	10	88
WR	Gansp	Agricomycotina	4	2	3	1	3	1	0	8	0	1	1	1	0	0	0	25	3	2	1	3	1	1	0	1	11	37
WR	Hetan	Agricomycotina	4	2	3	1	1	4	0	6	0	0	1	1	0	0	0	23	1	2	1	2	0		0	0	6	29
WR	Hetir	Agricomycotina	5	2	3	1	1	4	0	7	0	0	1	1	0	0	0	25	1	2	1	2	0	1	0	1	7	33
WR	Hypsu	Agricomycotina	13	5	2	1	2	7	0	14	0	0	1	1	0	0	0	46	2	3	2	1	1	1	0	1	10	57
WR	Jaaar	Agricomycotina	8	4	2		1	0	0	1	0	0	1	1	0	0	0	18	2	2	1	1	1	1	0	1	8	27
WR	Lenti	Agricomycotina	4	2	3	1	1	3	0	18	2	1	1	1	0	0	0	37	3	2	1	1	1	1	0	1	9	47
WR	Ompol	Agricomycotina	9	3	3	1	1	0	0	5	0	1	1	2	0	0	0	26	2	2	3	1	1	1	0	1	10	37
WR	Phach	Agricomycotina	3	3	5	1	0	0	10	5	0	1	1	1	0	0	0	30	2	2	2	1	1	1	0	1	9	40
WR	Phlbr	Agricomycotina	3	3	3	1	3	0	5	9	0	0	1	1	0	0	0	29	2	2	2	2	1	1	0	1	10	40
WR	Phlgi	Agricomycotina	4	4	2	1	5	0	3	5	0	1	1	1	0	0	0	27	2	2	2	1	1	1	0	1	9	37
WR	Pleos	Agricomycotina	4	2	2	1	4	0	0	5	4	0	1	1	0	0	0	24	2	1	3	2	1	1	0	1	10	35
WR	Pleos	Agricomycotina	4	4	2	1	4	0	0	5	4	0	1	1	0	0	0	26	2	1	3	2		1	0	1	9	36
WR	Plicr	Agricomycotina	3	3	1	1	0	0	0	7	0	0	1	1	0	0	0	17	3	2	1	2	1	1	0	1	10	28
WR	Polar	Agricomycotina	4	2	5	1	1	2	0	14	0	3	1	1	0	0	0	34	3	2	1	1	1	1	0	1	9	44
WR	Punst	Agricomycotina	8	2	2	1	5	1	0	11	0	0	1	1	0	0	0	32	2	2	1	1	1	1	0	2	8	42
WR	Schco	Agricomycotina	3	2	2	1	0	1	0	0	0	0	1	1	0	0	0	11	2	2	2	1	1	2	0	1	10	22
WR	Sissu	Agricomycotina	33	6	4	1	0	1	0	15	0	1	1	1	0	0	0	63	2	2	1	1	1	1	0	1	8	72

WR	Trave	Agricomycotina	3	2	3	1	2	0	11	14	1	0	1	1	0	0	0	39	3	2	1	1	1	1	0	1	9	49
WR	Stehi	Agricomycotina	10	3	3	1	2	1	0	6	0	0	1	2	0	0	0	29	1	3	1	3		1	0	1	9	39
WR	Strru	Agricomycotina	14	4	1	1	1	10	0	17	0	0	1	1	0	0	0	50	2	3	2	1	1	1	0	1	10	61
			7	3	3	1	2	1	1	5	0	0	1	1	0	0	0	25	2	2	1	1	1	1	0	1	9	35

Reference Name: Amamu: *Amanita muscaria* Koide; Hebcy: *Hebeloma cylindrosporum* h7; Lacam: *Laccaria amethystina* LaAM-08-12; Lacbi: *Laccaria bicolor*; Paxin: *Paxillus involutus* ATCC 200175; Paxru: *Paxillus rubicundulus* Ve08.2h10; Pilcr: *Piloderma croceum* F 1598; Pismi: *Pisolithus microcarpus* 441; Pisti: *Pisolithus tinctorius* Marx 270; Pirin: *Piriformospora indica* DSM 11827; Sclci: *Scleroderma citrinum* Foug A; Suibr: *Suillus brevipes* Sb2 2; Suilu: *Suillus luteus* UH-Slu-Lm8-n1; Sebve: *Sebacina vermifera* MAFF 305830; Treme: *Tremella mesenterica*; Tulca: *Tulasnella calospora* AL13; Calco: *Calocera cornea*; Calvi: *Calocera viscosa*; Conpu: *Coniophora puteana*; Dacsp: *Dacryopinax* sp. DJM 731 SSP1; Daequ: *Daedalea quercina*; Fishe: *Fistulina hepatica*; Fompi: *Fomitopsis pinicola* FP-58527 SS1; Glotr: *Gloeophyllum trabeum*; Hydpi: *Hydnomerulius pinastri*; Laesu: *Laetiporus sulphureus* var. sulphureus; Neole: *Neolentinus lepideus*; Pospl: *Postia placenta* 1; Serla: *Serpula lacrymans* S7.3; Wolco: *Wolfiporia cocos*; Agabi: *Agaricus bisporus*; Amath: *Amanita thiersii* Skay4041; Copci: *Coprinopsis cinerea* okayama7; Gymlu: *Gymnopus luxurians*; Aursu: *Auricularia subglabra*; Bjead: *Bjerkandera adusta* 1; Botbo: *Botryobasidium botryosum*; Cylto: *Cylindrobasidium torrendii*; Dicsq: *Dichomitus squalens* ; Exigl: *Exidia glandulosa*; Fibsp: *Fibulorhizoctonia* sp. CBS 109695; Fomme: *Fomitiporia mediterranea*; Galma: *Galerina marginata*; Gansp: *Ganoderma* sp. 10597 SS1; Hetan: *Heterobasidion annosum* 03012 (FBL); Hetir: *Heterobasidion irregulare* TC 32-1; Hypsu: *Hypholoma sublateritium*; Jaaar: *Jaapia argillacea*; Lenti: *Lentinus tigrinus*; Ompol: *Omphalotus olearius*; Phach: *Phanerochaete chrysosporium* 2.1; Phlbr: *Phlebia brevispora* HHB-7030 SS6; Phlgi: *Phlebiopsis gigantea* 1; Pleos: *Pleurotus ostreatus* PC15; Pleos: *Pleurotus ostreatus* PC9; Plicr: *Plicaturopsis crispa*; Polar: *Polyporus arcularius*; Punst: *Punctularia strigosozonata*; Schco: *Schizophyllum commune* H4-8; Sissu: *Sistotremastrum suecicum*; Trave: *Trametes versicolor*; Stehi: *Stereum hirsutum* FP-91666 SS1; Strru: *Stropharia rugosoannulata*

Table S10 17 MnP genes classification

	trp171	Glu35	Glu39	Asp179	
	W	E	E	D	
**Gene ID**	**Wc**	**Mn Ea**	**Mn Eb**	**Mn Dc**	**Classification**
SRUG_03862	-	D	-	+	MnP atypical
SRUG_03871	-	D	-	+	MnP atypical
SRUG_03872	-	D	-	+	MnP atypical
SRUG_04745	-	D	-	+	MnP atypical
SRUG_07844	-	D	-	+	MnP atypical
SRUG_07846	-	D	-	+	MnP atypical
SRUG_07847	-	D	-	+	MnP atypical
SRUG_07849	-	D	-	+	MnP atypical
SRUG_07850	-	D	-	+	MnP atypical
SRUG_07851	-	D	-	+	MnP atypical
SRUG_08480	-	+	-	-	MnP atypical
SRUG_09291	-	+	-	+	MnP atypical
SRUG_09292	-	+	-	+	MnP atypical
SRUG_09293	-	+	-	+	MnP atypical
SRUG_09779	-	D	-	+	MnP atypical
SRUG_09835	-	D	-	+	MnP atypical
SRUG_10902	-	-	-	+	MnP atypical

Table S11 Putative peroxidase-encoding genes of *Stropharia rugosoannulata*

Gene ID	Classfication	Gene expression			secretory protein
SRUG_01340	Haloperoxidase(haem)	0	0	0	Y
SRUG_01341	Haloperoxidase(haem)	0	0	0	Y
SRUG_01346	Haloperoxidase(haem)	0	0.051446	0	Y
SRUG_02748	Haloperoxidase(haem)	2.650975	1.244453	6.433436	N
SRUG_06741	Haloperoxidase(haem)	34.16175	20.35598	22.5075	Y
SRUG_06742	Haloperoxidase(haem)	0.090192	0.105197	0.2741	Y
SRUG_06743	Haloperoxidase(haem)	0.102564	0.105836	0.054406	Y
SRUG_09264	Haloperoxidase(haem)	0.033723	0	0	Y
SRUG_09697	Haloperoxidase(haem)	100.6336	150.6177	106.1437	N
SRUG_09851	Haloperoxidase(haem)	0	0.151478	0.178881	Y
SRUG_10620	Haloperoxidase(haem)	9.229263	8.807705	4.983132	Y
SRUG_10678	Haloperoxidase(haem)	34.3484	46.3467	38.22605	N
SRUG_11079	Haloperoxidase(haem)	4.528346	3.706427	8.901436	N
SRUG_02749	Haloperoxidase(haem)	0	0.322391	0	N
SRUG_00066	Linoleate diol synthase	7.362726	11.05866	8.1958	N
SRUG_05824	Linoleate diol synthase	4.074601	9.285253	5.951723	N
SRUG_06857	Linoleate diol synthase	13.65838	30.18056	20.8284	N
SRUG_08651	Linoleate diol synthase	127.2217	132.7035	376.1703	N
SRUG_06581	Catalase	15.82494	19.61318	23.73195	N
SRUG_01177	Cytochrome-c peroxidase	76.43199	80.94376	74.81068	N
SRUG_08269	DyP-type peroxidase D	20.09621	22.44544	54.79304	N
SRUG_03814	Hybrid Ascorbate-Cytochrome C peroxidase	0.995445	2.164737	1.316597	Y
SRUG_02817	Hybrid Ascorbate-Cytochrome C peroxidase	322.7769	405.7787	222.0444	Y
SRUG_04040	Hybrid Ascorbate-Cytochrome C peroxidase	0	0.037536	0.039402	Y

SRUG_04753	Hybrid Ascorbate-Cytochrome C peroxidase	0.040854	0.063005	0	Y
SRUG_06801	Hybrid Ascorbate-Cytochrome C peroxidase	0.905026	1.007475	2.030694	Y
SRUG_07015	Hybrid Ascorbate-Cytochrome C peroxidase	12.11928	22.69461	16.07476	Y
SRUG_07017	Hybrid Ascorbate-Cytochrome C peroxidase	0	0	0.02872	Y
SRUG_07052	Hybrid Ascorbate-Cytochrome C peroxidase	12.26524	17.4184	15.09075	Y
SRUG_08368	Hybrid Ascorbate-Cytochrome C peroxidase	18.48481	29.51112	22.87286	Y
SRUG_09055	Hybrid Ascorbate-Cytochrome C peroxidase	0.094905	0.603982	0.730356	Y
SRUG_03862	Manganese peroxidase	0.159296	0.138953	0.058596	Y
SRUG_03871	Manganese peroxidase	21.65204	15.61914	18.20432	N
SRUG_03872	Manganese peroxidase	32.80987	12.34284	41.7977	Y
SRUG_04745	Manganese peroxidase	0.096936	0	0	Y
SRUG_07847	Manganese peroxidase	6.734917	29.52272	35.65636	N
SRUG_07850	Manganese peroxidase	70.89768	34.65069	65.00932	Y
SRUG_07851	Manganese peroxidase	7.884882	8.216792	6.24357	Y
SRUG_08480	Manganese peroxidase	0.593793	11.54898	1.837162	N
SRUG_09291	Manganese peroxidase	0	0	0	Y
SRUG_09292	Manganese peroxidase	0	0	0	Y
SRUG_09293	Manganese peroxidase	0.106754	0.739416	0	Y
SRUG_09779	Manganese peroxidase	0.223447	1.113872	0.204948	Y
SRUG_09835	Manganese peroxidase	0	0.15611	0	Y
SRUG_10902	Manganese peroxidase	11.61757	10.51701	9.184996	N
SRUG_07844	Manganese peroxidase	0.235738	1.498493	0.342125	Y
SRUG_07846	Manganese peroxidase	25.95676	28.84586	19.17442	Y
SRUG_07849	Manganese peroxidase	20.09236	35.52051	26.72743	Y
SRUG_01647	NoxA	1758.868	1433.841	1889.156	N
SRUG_09509	NoxB	184.4253	250.9619	216.5775	N

SRUG_00593	NoxR	393.837	333.0223	403.0918	N
SRUG_06711	Atypical 2-Cysteine peroxiredoxin (typeII, typeV)	321.418	228.841	175.491	N
SRUG_08102	Atypical 3-Cysteine peroxiredoxin (typeII, typeV)	49.40612	46.57563	65.07483	N
SRUG_11529	Atypical 2-Cyste(typeQ, BCP)	4462.049	3478.453	3798.053	N
SRUG_07277	Atypical 3-Cyste(typeQ, BCP)	244.0841	287.9891	319.1688	N
SRUG_06701	Atypical 4-Cyste(typeQ, BCP)	62.72382	41.96613	50.8286	N
SRUG_05238	1-Cysteine peroxiredoxin (1CysPrx)	431.7512	747.7196	843.8557	N
SRUG_04690	1-Cysteine peroxiredoxin (2CysPrx)	149.7155	302.9699	238.3941	N
SRUG_06761	Carboxymuconolactone decarboxylase	40.97107	22.10519	28.68509	N
SRUG_04254	Fungi-Bacteria glutathione peroxidase	521.6467	376.27	810.6085	N
SRUG_08181	Typical 2-Cysteine peroxiredoxin	10.75503	27.96025	10.66499	N

Table S12 The number of CAZyme families in 16 species

Specise	GT	GH	CE	PL	AA	CBM	Summary
Armillaria mellea	63	209	33	22	103	9	439
Agaricus bisporus	50	144	18	19	70	4	305
Coprinopsis cinerea	59	153	32	15	81	9	349
Hypholoma sublateritium	61	163	23	8	98	7	360
Lepista nuda	62	217	33	24	108	10	454
Lyophyllum atratum	67	206	23	13	104	5	418
Lentinula edodes	51	155	10	6	47	5	274
Laccaria bicolor	54	98	12	6	31	1	202
Moniliophthora roreri	62	240	36	23	118	9	488
Pholiota highlandensis	63	177	25	10	121	8	404
Psilocybe cubensis	58	182	23	8	105	21	397
Pleurotus eryngii	57	181	19	24	84	9	374
Pluteus cervinus	58	167	18	10	78	6	337
Schizophyllum commune	61	182	19	17	51	3	333
Stropharia rugosoannulata	55	165	25	10	107	9	371

Table S13-1 Comparative analysis of the plant cell wall degradation (PCWD) gene families with 37 fungi — Cellulose

			Cellulose												
**Nutrtional strategy**	**Order**	**Species acronym**	**GH7**	**GH6**	**AA9 LPMO**	**GH12**	**GH5-5**	**GH3 b-gluc**	**GH1**	**ALE**	**GH9**	**CDH**	**AA8 cytb6 52**	**AA12**	**Total cellulose related genes**
WR	Auriculariales	Aursu	6	2	19	2	4	12	1	4	0	1	0	0	51
WR	Polyporales	Bjead	5	1	26	2	4	8	2	3	1	1	1	0	54
WR	Polyporales	Dicsq	3	1	14	3	3	6	4	5	0	1	1	0	41
WR	Auriculariales	Exigl	6	1	34	2	7	11	1	5	0	1	0	0	68
WR	Hymenochaetales	Fomme	2	2	13	3	3	8	5	3	1	1	0	0	41
WR	Polyporales	Cersu	3	1	9	2	2	5	3	2	0	1	1	0	29
WR	Agaricales	Gymlu	7	1	12	3	4	12	3	3	1	1	0	0	47
WR	Agaricales	Galma	8	3	18	4	7	9	5	5	1	1	0	0	61
WR	Polyporales	Gansp	3	1	15	3	3	8	3	5	1	1	1	0	44
WR	Amylocorticiales	Plicr	1	2	9	2	2	9	3	5	1	1	1	0	36
WR	Polyporales	Phlbr	4	1	12	2	4	7	2	3	1	1	1	0	38
WR	Corticiales	Punst	5	1	13	2	3	13	1	4	1	1	0	0	44
WR	Russulales	Peni	3	1	16	4	5	8	1	4	1	1	0	0	44
WR	Trechisporales	Sissu	3	4	21	3	6	7	1	2	0	2	1	0	50
WR	Trechisporales	Sisni	3	4	21	2	6	7	1	2	0	2	1	0	49
WR	Russulales	Stehi	3	1	16	5	4	14	3	4	1	1	1	0	53
WR	Polyporales	Trave	4	1	18	5	3	10	2	3	1	1	1	0	49
LD	Agaricales	Agabi	1	1	10	2	3	5	1	3	1	1	0	0	28

LD	Agaricales	AGP	7	3	19	2	5	7	3	4	1	1	0	0	52
LD	Agaricales	Copci	6	5	32	0	1	7	2	3	1	1	0	4	62
LD	Agaricales	COA	5	2	38	0	2	6	2	3	1	1	0	2	62
LD	Agaricales	GYC	5	1	10	3	4	11	3	3	1	1	0	0	42
LD	Agaricales	LEL	7	2	23	3	4	11	3	4	1	4	0	0	62
LD	Agaricales	PSS	7	3	26	5	9	8	4	3	1	1	2	0	69
LD	Agaricales	TEN	4	1	14	4	2	18	4	4	1	1	0	0	53
LD	Agaricales	TRC	6	2	23	3	2	8	2	3	1	2	0	0	52
BR	Dacrymycetales	Calvi	0	0	0	1	2	11	1	4	1	0	0	0	20
BR	Dacrymycetales	Calco	0	0	0	1	2	9	1	4	1	0	0	0	18
BR	Boletales	Conpu	2	2	10	4	5	10	3	4	1	2	3	0	46
BR	Dacrymycetales	Dacsp	0	0	0	1	3	7	1	3	1	0	0	0	16
BR	Polyporales	Daequ	0	0	4	2	2	6	2	3	0	0	0	0	19
BR	Polyporales	Fompi	0	0	4	2	3	9	2	3	0	0	0	0	23
BR	Gloeophyllales	Glotr	0	0	4	2	2	9	5	4	1	1	0	0	28
BR	Boletales	Hydpi	3	1	14	3	5	11	3	3	1	1	3	0	48
BR	Gloeophyllales	Neole	0	0	3	2	2	7	2	4	1	0	0	0	21
BR	Boletales	Serla 7.3	0	1	5	1	3	9	3	3	1	2	2	0	30
BR	Polyporales	Wolco	0	0	2	2	2	6	1	2	0	0	0	0	15
LD/WR	Strophariaceae	Strru	4	1	11	1	3	7	3	2	1	1	1	0	35

Table S13-2 Comparative analysis of the PCWD gene families with 37 fungi — Hemicellulose

	**Hemicellulose**

						xylan								pectin					mannan				xyloglucan		cutin	
**Nutrtional strategy**	**Order**	**Species acronym**	**GH10**	**GH11**	**GH3 b-xylo**	**CE1**	**CE16**	**GH115**	**GH62**	**GH28**	**CE12**	**CE8**	**GH35**	**GH53**	**GH43**	**GH78**	**GH88**	**GH5-7**	**GH2**	**GH27**	**GH74**	**GH51**	**GH95**	**GH29**	**CE5**	**Total hemicellulose related genes**
WR	Auriculariales	Aursu	4	3	1	4	31	1	0	10	2	3	6	1	27	4	2	2	7	4	1	3	1	2	3	122
WR	Polyporales	Bjead	4	0	0	1	13	2	0	6	1	2	4	1	6	2	1	2	3	3	2	2	1	0	0	56
WR	Polyporales	Dicsq	5	0	1	0	11	2	0	7	2	3	3	1	7	5	1	2	4	6	1	2	1	0	0	64
WR	Auriculariales	Exigl	8	2	1	6	29	2	2	12	4	4	7	1	22	6	3	2	6	10	1	4	1	3	2	138
WR	Hymenochaetales	Fomme	4	0	0	0	6	3	0	14	2	3	2	1	6	2	2	2	2	4	4	1	2	0	0	60
WR	Polyporales	Cersu	6	1	0	1	7	2	0	6	0	2	1	5	2	1	1	2	4	4	1	2	1	0	0	49
WR	Agaricales	Gymlu	5	4	2	5	12	1	1	19	3	6	5	1	9	3	1	5	4	6	1	5	2	1	3	104
WR	Agaricales	Galma	8	8	1	2	10	1	1	17	4	3	9	3	4	1	1	8	3	8	2	5	2	1	6	108
WR	Polyporales	Gansp	8	0	4	2	17	2	0	10	1	2	6	1	11	4	1	2	3	3	1	2	2	0	0	82
WR	Amylocorticiales	Plicr	2	0	0	4	11	1	0	10	0	3	2	0	3	3	1	2	4	2	1	2	2	1	0	54
WR	Polyporales	Phlbr	8	0	0	1	8	2	0	5	0	3	4	1	1	1	1	2	2	2	1	1	2	1	1	47
WR	Corticiales	Punst	5	1	0	2	12	1	0	12	1	6	4	2	7	7	1	1	4	5	2	3	1	1	1	79
WR	Russulales	Peni	6	1	1	2	12	1	0	13	4	4	5	1	19	2	1	2	2	5	1	4	3	2	3	94
WR	Trechisporales	Sissu	5	2	0	3	4	2	0	2	0	0	1	0	2	0	0	3	2	1	2	1	1	0	2	33
WR	Trechisporales	Sisni	5	2	0	3	5	2	0	2	0	0	1	0	2	0	0	5	2	1	2	1	1	0	2	36
WR	Russulales	Stehi	6	1	2	1	11	2	0	17	3	4	7	2	10	3	1	1	3	5	2	3	1	4	1	90
WR	Polyporales	Trave	6	0	2	3	7	2	0	9	0	2	2	1	3	3	1	2	5	4	1	2	1	0	0	56
LD	Agaricales	Agabi	2	2	2	1	11	2	0	5	2	2	1	1	4	4	1	1	2	4	1	1	1	1	6	57
LD	Agaricales	AGP	8	3	1	2	6	1	2	7	2	3	4	1	3	1	1	3	2	4	1	3	1	0	4	63

LD	Agaricales	Copci	5	6	0	3	5	1	3	3	1	0	0	1	4	0	1	2	2	0	1	1	0	0	6	45
LD	Agaricales	COA	18	5	1	5	3	1	1	4	1	0	2	1	5	0	1	3	2	1	1	1	0	0	2	58
LD	Agaricales	GYC	3	3	2	2	3	1	1	15	5	8	3	1	8	2	1	4	4	5	1	4	2	1	6	85
LD	Agaricales	LEL	5	1	2	1	5	2	1	7	3	4	5	2	9	4	1	4	3	8	1	2	2	1	4	77
LD	Agaricales	PSS	10	12	1	5	5	1	4	7	1	5	4	1	10	1	1	7	2	6	1	4	2	0	10	100
LD	Agaricales	TEN	5	3	2	2	13	2	1	14	4	7	7	1	8	3	1	2	4	5	1	4	3	3	5	100
LD	Agaricales	TRC	3	0	1	2	3	2	0	6	2	2	5	1	7	3	1	3	3	5	1	4	1	1	5	61
BR	Dacrymycetales	Calvi	2	1	1	0	4	2	0	6	0	2	1	1	4	0	2	2	4	2	0	2	0	2	0	38
BR	Dacrymycetales	Calco	2	2	1	0	5	2	0	7	0	2	1	1	4	0	1	2	1	2	0	2	0	2	0	37
BR	Boletales	Conpu	3	0	2	0	7	2	0	13	0	2	2	1	6	2	1	2	5	3	0	3	1	4	1	60
BR	Dacrymycetales	Dacsp	3	0	1	0	4	2	0	6	0	2	1	1	5	0	1	2	3	2	0	2	0	2	0	37
BR	Polyporales	Daequ	3	0	2	1	7	1	0	8	0	1	1	1	3	3	1	2	3	3	0	1	1	0	0	42
BR	Polyporales	Fompi	3	0	2	0	9	1	0	12	0	2	2	1	7	3	1	2	4	4	0	3	1	0	0	57
BR	Gloeophyllales	Glotr	3	0	1	1	7	2	0	10	0	2	2	2	5	2	1	2	4	3	1	3	1	1	0	53
BR	Boletales	Hydpi	3	0	1	1	10	1	0	9	0	2	2	1	2	2	1	3	3	6	1	1	1	1	0	51
BR	Gloeophyllales	Neole	2	0	1	0	3	1	0	10	0	2	2	1	4	1	1	2	3	3	1	3	1	1	0	42
BR	Boletales	Serla 7.3	1	0	1	0	3	1	0	7	0	2	3	1	1	2	1	4	3	3	1	1	1	1	0	37
BR	Polyporales	Wolco	4	0	1	0	5	2	0	9	0	1	2	1	1	3	1	2	3	3	0	4	1	0	0	43
LD/WR	Strophariaceae	Strru	4	1	1	3	4	1	0	8	1	4	3	1	4	2	1	2	3	2	1	8	2	0	5	61

Table S13-3 Comparative analysis of the PCWD gene families with 37 fungi — Lignin and xenobiotics

			Lignin and xenobiotics

Nutrtional strategy	Order	Species acronym	AA2 ligninolytic POD	AA1 Lac and Lac-like	DyP	HTP	AA5 GLX	AA5 CRO other	AA3_3 GMC MOX	AA3_2 GMC PDH,GO, AAOX	AA3_4 PO	Total lignin and xenobiotics related genes	Total copies per species
WR	Auriculariales	Aursu	5	6	11	16	2	6	5	33	3	87	260
WR	Polyporales	Bjead	18	1	11	4	1	5	7	28	1	76	186
WR	Polyporales	Dicsq	12	13	1	4	5	4	4	29	0	72	177
WR	Auriculariales	Exigl	13	7	8	32	2	6	5	34	4	111	317
WR	Hymenochaetales	Fomme	16	10	3	4	0	4	3	22	0	62	163
WR	Polyporales	Cersu	14	8	0	9	0	3	4	17	0	55	133
WR	Agaricales	Gymlu	5	17	12	17	5	5	9	48	0	118	269
WR	Agaricales	Galma	17	8	5	24	4	12	4	31	0	105	274
WR	Polyporales	Gansp	8	16	3	4	5	4	5	28	0	73	199
WR	Amylocorticiales	Plicr	6	5	0	3	0	5	4	14	0	37	127
WR	Polyporales	Phlbr	13	8	3	3	1	6	6	31	1	72	157
WR	Corticiales	Punst	10	12	5	8	3	6	4	17	1	66	189
WR	Russulales	Peni	14	21	0	2	1	6	2	32	1	79	217
WR	Trechisporales	Sissu	14	13	0	35	0	6	1	22	2	93	176
WR	Trechisporales	Sisni	14	13	0	35	0	6	1	21	2	92	177
WR	Russulales	Stehi	5	18	2	9	3	5	7	41	0	90	233
WR	Polyporales	Trave	25	8	2	3	5	4	4	18	1	70	175
LD	Agaricales	Agabi	2	11	0	24	3	6	4	29	0	79	164
LD	Agaricales	AGP	7	11	2	4	3	11	4	24	0	66	181
LD	Agaricales	Copci	0	17	3	8	0	6	2	32	0	68	175

LD	Agaricales	COA	0	15	1	14	0	6	2	30	0	68	188
LD	Agaricales	GYC	6	21	14	18	2	4	5	31	0	101	228
LD	Agaricales	LEL	2	19	1	11	5	6	2	56	0	102	241
LD	Agaricales	PSS	9	7	3	13	5	8	4	28	0	77	246
LD	Agaricales	TEN	9	22	7	27	9	22	7	70	1	174	327
LD	Agaricales	TRC	1	24	1	4	1	5	1	16	0	53	166
BR	Dacrymycetales	Calvi	0	6	0	6	0	3	1	9	0	25	83
BR	Dacrymycetales	Calco	0	3	0	7	0	3	2	13	0	28	83
BR	Boletales	Conpu	0	7	0	2	0	6	4	15	0	34	140
BR	Dacrymycetales	Dacsp	0	3	0	6	0	3	1	8	0	21	74
BR	Polyporales	Daequ	0	4	0	4	0	2	4	13	0	27	88
BR	Polyporales	Fompi	0	6	0	4	0	3	5	22	0	40	120
BR	Gloeophyllales	Glotr	0	4	0	6	0	2	2	20	1	35	116
BR	Boletales	Hydpi	0	10	1	4	0	5	4	7	1	32	131
BR	Gloeophyllales	Neole	0	4	0	9	0	2	3	21	0	39	102
BR	Boletales	Serla 7.3	0	5	0	3	0	3	5	7	0	23	90
BR	Polyporales	Wolco	0	4	0	5	0	4	5	9	0	27	85
LD/WR	Strophariaceae	Strru	17	10	1	14	10	1	8	37	0	98	194

Aursu: *Auricularia subglabra*; Bjead: *Bjearkandera adusta*; Dicsq: *Dichomitus squalens*; Exigl: *Exidia glandulosa*; Fomme: *Fomitiporia meditteranea*; Cersu: *Gelatopria subvermispora*; Gymlu: *Gymnopus luxurians*; Galma: *Galerina marginata*; Gansp: *Ganoderma* sp.; Plicr: *Plicaturposis crispa*; Phlbr: *Phlebia brevispora*; Punst: *Punctularia strigoso-zonata*; Peni: *Peniophora* sp.; Sissu: *Sistotremastrum suecicum*; Sisni: *Sistotremastrum niveocremeum*; Stehi: *Stereum hirsutum*; Trave: *Trametes versicolor*; Agabi: *Agaricus bisporus* var. bisporus; AGP: *Agrocybe pediades*; Copci: *Coprinopsis cinerea*; COA: *Coprinellus angulatus*; GYC: *Gymnopus confluens*; LEL: *Leucoagaricus leucothites*; PSS: *Psilocybe* cf. *subviscida*; TEN: *Tetrapyrgos nigripes*; TRC: *Tricholomella constricta*; Calvi: *Calocera viscosa*; Calco: *Calocera cornea*; Conpu: *Coniophora puteana*; Dacsp: *Dacryopinax spathulata*; Daequ: *Daedalea quercina*; Fompi: *Fomitopsis pinicola*; Glotr: *Gloeophyllum trabeum*; Hydpi: *Hydnomeruliu pinastri*; Neole: *Neolentinus lepideus*; Serla 7.3: *Serpula lacrymans*; Wolco: *Wolfiporia cocos*; Strru: *Stropharia rugosoannulata*

Table S14-1 Variables Failing Tolerance Test

	Within-Groups Variance	Tolerance	Minimum Tolerance
CE5	2.442	0.000	0.000
AA2 ligninolytic POD	16.986	0.000	0.000
AA1 Lac and Lac-like	21.923	0.000	0.000
DyP	12.85	0.000	0.000
HTP	83.563	0.000	0.000
AA5 GLX	3.864	0.000	0.000
AA5 CRO other	9.47	0.000	0.000
AA3_3GMC MOX	3.604	0.000	0.000
AA3_2 GMC PDH,GO, AAOX	115.527	0.000	0.000
AA3_4 PO	0.749	0.000	0.000

All variables passing the tolerance criteria are entered simultaneously.

a. Minimum tolerance level is .001.

Table S14-2 Classification function coefficients resulting from discriminant analysis obtained from PCWD characters

	Classification Function Coefficients		
	BR	LD	WR
GH7	21.505	-121.525	-29.628
GH6	-104.222	244.885	17.089
AA9 LPMO	-12.887	35.998	2.238
GH12	10.678	202.69	105.75
GH5-5	53.545	-74.52	6.064
GH3 b-gluc	18.464	-53.72	-16.901
GH1	-35.09	39.184	-41.726
ALE	82.83	-92.059	54.707
GH9	-91.465	75.512	-52.679
CDH	-16.862	173.085	66.933
AA8 cytb652	51.006	-222.112	-28.949
AA12	122.179	63.105	127.942
GH10	-3.115	23.333	5.792
GH11	-33.087	-32.848	-36.6
GH3 b-xylo	-56.661	118.358	-31.206
CE1	24.026	-57.162	17.712
CE16	-3.083	65.9	24.241
GH115	72.131	-196.624	35.503
GH62	14.025	-191.225	-63.856
GH28	16.254	-46.295	5.287
CE12	-5.513	-90.499	-28.487
CE8	-55.672	316.139	94.223
GH35	-4.233	-25.553	8.533
GH53	35.532	12.378	43.073
GH43	6.341	-40.861	-10.546
GH78	12.607	-65.157	-25.489
GH88	-21.523	365.725	87.295
GH5-7	48.952	81.61	62.867
GH2	-2.749	-120.508	-38.858
GH27	-33.739	-0.087	-31.123
GH74	47.841	-84.204	6.82
GH51	-9.407	124.047	8.809
GH95	13.514	-214.703	-87.56
GH29	-24.64	11.537	-50.174
(Constant)	-245.397	-374.766	-323.187

Fisher's linear discriminant functions

Table S14-3 Classification results[a]

			Predicted Group Membership			Total
			BR	LD	WR	
Original	Count	BR	11	0	0	11
		LD	0	9	0	9
		WR	0	0	17	17
		Ungrouped cases (SR)	0	1	0	1
	%	BR	100	0	0	100
		LD	0	100	0	100
		WR	0	0	100	100
		Ungrouped cases (SR)	0	100	0	100

a.100.0% of original grouped cases correctly classified.

Table S15 The gene clusters of *Stropharia rugosoannulata* predicted by antiSMASH

Region	Type	From	To
Region 1.1	NRPS-like	4,035,697	4,079,731
Region 2.1	NRPS-like	338,290	388,136
Region 2.2	NRPS-like	408,253	464,478
Region 2.3	siderophore	3,397,577	3,409,795
Region 3.1	terpene	666,283	687,725
Region 3.2	NRPS	1,783,703	1,831,735
Region 4.1	siderophore	1,960,289	1,972,449
Region 4.2	terpene	2,395,330	2,416,595
Region 4.3	NRPS-like	3,572,551	3,616,679
Region 5.1	terpene	1,053,109	1,074,431
Region 6.1	terpene	340,380	355,479
Region 6.2	terpene	647,505	679,621
Region 6.3	terpene	2,077,902	2,099,118
Region 7.1	terpene	465,221	485,608
Region 7.2	NRPS-like	1,626,863	1,674,587
Region 7.3	terpene	2,536,998	2,555,968
Region 7.4	terpene	2,567,855	2,589,139
Region 8.1	NRPS-like	239,884	283,810
Region 8.2	terpene	1,148,201	1,164,861
Region 8.3	terpene	2,224,009	2,245,487
Region 8.4	T1PKS	2,256,646	2,303,282
Region 9.1	NRPS-like	233,391	255,448
Region 9.2	terpene	1,580,441	1,600,518
Region 9.3	terpene	2,160,936	2,182,205
Region 9.4	NRPS-like,T1PKS	2,562,731	2,610,976
Region 10.1	NRPS-like	2,251,608	2,297,150
Region 10.2	terpene	2,384,642	2,406,483
Region 11.1	NRPS-like	1,656,079	1,700,094
Region 12.1	indole	679,975	698,519
Region 12.2	terpene	1,482,858	1,503,883
Region 12.3	indole	1,656,043	1,677,263
Region 12.4	NRPS-like	2,124,080	2,168,664
Region 15.1	terpene	1,153,025	1,174,185
Region 16.1	indole	513,075	534,267

Table S16 The number of gene clusters predicted by antiSMASH

Speies name	NRPS like	NRPS	terpene	siderophone	T1PKS	indole	others	total
Agrocybe praecox	8	1	12	1	2	2	2	28
Armillaria mellea	13	1	22	2	4	0	4	46
Agaricus bisporus	6	0	10	1	1	2	0	20
Coprinopsis cinerea	2	1	9	1	2	1	0	16
Ganoderma sinense	9	1	20	0	3	0	1	34
Hypholoma sublateritium	5	1	12	1	1	0	2	22
Lyophyllum atratum	9	0	10	1	1	1	0	22
Lepista nuda	12	1	24	1	2	1	5	46
Lentinula edodes	5	2	10	0	4	0	0	21
Moniliophthora roreri	10	2	24	0	9	3	1	39
Pholiota highlandensis	10	0	14	1	3	2	2	32
Psilocybe cubensis	6	1	21	1	1	0	2	32
Pholiota alnicola	11	0	22	1	2	0	2	38
Pleurotus eryngii	4	0	12	1	0	0	3	20
Pluteus cervinus	5	1	13	1	1	0	0	21
Schizophyllum commune	5	4	5	0	1	0	1	16
Stropharia rugosoannulata	10	1	16	2	1	3	1	34

Table S17 NRPS gene cluster responsible for coprinoferrin in *Coprinopsis cinerea*, *Stropharia rugosoannulata* and *Hypholoma sublateritium*

Gene ID	Proposed protein function	Homologous Gene ID	Identity (%)	E-value	Homologous Gene ID	Identity (%)	E-value
CC1G_04201	Papain inhibitor	SRUG_03244	44.44	4.00E-57	HYPSUDRAFT_630361	60.63	3.00E-111
CC1G_04202	Secretory carrier-associated membrane protein	SRUG_03245	50.22	6.00E-72	HYPSUDRAFT_179320	79.1	3.00E-137
CC1G_04203	Polyadenylate-binding protein	SRUG_03246	76.08	0	HYPSUDRAFT_35211	91.52	0
CC1G_04204	Methyltransferases lae 1	SRUG_03247	77.42	4.00E-51	HYPSUDRAFT_198131	83.39	0
CC1G_04205	Transcription elongation factor spt6	SRUG_03248	44.62	2.00E-26	HYPSUDRAFT_35206	73.15	1.00E-49
CC1G_04207	Transcription factor SPT8	SRUG_03249	50.87	2.00E-95	HYPSUDRAFT_35205	71.46	0
CC1G_04208	Transcription elongation factor SPT6	SRUG_03250	61.75	0	HYPSUDRAFT_35204	82.55	0
CC1G_04209	Diaminohydroxyphosphoribosylamino-pyrimidine deaminase	SRUG_03251	60.84	2.00E-68	HYPSUDRAFT_131028	70	2.00E-83
CC1G_04210	Nonribosomal peptide synthase	SRUG_03252	44.8	0	HYPSUDRAFT_63026	61.06	0
CC1G_04211	Lysine/ornithine N-monooxygenase	SRUG_03253	57.27	0	HYPSUDRAFT_51897	69.51	0
CC1G_10829	/	SRUG_03254	42.55	3.00E-75	HYPSUDRAFT_63024	46.26	3.00E-130
CC1G_04212	Ribosome biogenesis protein RLP24	SRUG_03255	83.24	3.00E-107	HYPSUDRAFT_179299	89.89	3.00E-11
CC1G_04213	Autophagy-related protein 2	SRUG_03256	46.39	0	HYPSUDRAFT_63022	60.68	0.00E+00
CC1G_04214	SGNH_hydrolase/acetylesterase	-	-	-	HYPSUDRAFT_35643	-	-
CC1G_13904	Replication factor A protein 2	SRUG_03465	33.78	1.00E-16	HYPSUDRAFT_35641	34.84	3.00E-18
CC1G_13905	Replication factor A protein 2	SRUG_03477	53.82	2.00E-93	HYPSUDRAFT_35641	80.74	8.00E-150
CC1G_04216	N-acetyltransferase	SRUG_03478	58.17	0	HYPSUDRAFT_35639	78.74	0

Gene ID	Proposed protein function	Homologous Gene ID	Identity (%)	E-value	Homologous Gene ID	Identity (%)	E-value
CC1G_14430	ABC protein	SRUG_04292	54.32	0	HYPSUDRAFT_36540	78.77	0
CC1G_13741	Methylcrotonoyl-CoA carboxylase subunit alpha, mitochondrial	SRUG_02401	72.86	0	HYPSUDRAFT_82913	80.69	0
CC1G_07067	Ferric iron reductase FhuF-like transporter	SRUG_02402	60.14	0	HYPSUDRAFT_132979	73.69	0
CC1G_07068	Ferric iron reductase FhuF-like transporter	SRUG_04293	48.43	2.00E-158	HYPSUDRAFT_36546	73.06	0
CC1G_07069	/	SRUG_04294	31.97	1.00E-70	HYPSUDRAFT_159226	48.17	2.00E-146

Table S18 P450 genes involved in xenobiotic metabolism in *Stropharia rugosoannulata*

query	subject	identify	evalue	Nelson's P450 Name	identity	Blast e value	Putative functions
SRUG_00066	estExt_Genewise1.C_500019	82.794	0	CYP5068B1	22.80%	6.00E-26	Xenobiotic metabolism
SRUG_00694	estExt_Genewise1Plus.C_550131	76.425	0	CYP532A3	82.55%	0	Xenobiotic metabolism
SRUG_00967	fgenesh1_pm.6_#_128	83.818	0	CYP530A5c	49.33%	1.00E-32	Xenobiotic metabolism
SRUG_01555	EEB90551.1	61.983	3.90E-58	CYP620H1	61.42%	0	Xenobiotic metabolism
SRUG_01356	estExt_Genewise1Plus.C_1250038	78.968	0	CYP5080B3b	100.00%	0	Xenobiotic metabolism
SRUG_03658	fgenesh1_kg.5_#_482_#_Locus1473v6rpkm0.35_PRE	55.738	0	CYP584E2b	38.74%	3.00E-99	Xenobiotic metabolism
SRUG_03479	HCB03452.1	44.14	0	CYP620B1	24.74%	5.00E-34	Xenobiotic metabolism
SRUG_03956	fgenesh1_kg.109_#_10_#_Locus6691v4rpkm0.14_PRE	69.579	2.60E-164	CYP532A6	49.18%	1.00E-140	Xenobiotic metabolism
SRUG_04349	e_gw1.165.1.1	83.217	0	CYP58D3ab	100.00%	1.00E-107	Xenobiotic metabolism
SRUG_04713	fgenesh1_kg.23_#_203_#_Locus4066v1rpkm29.98	45.924	3.43E-153	CYP677A1	47.88%	1.00E-128	Xenobiotic metabolism
SRUG_05741	fgenesh1_pm.41_#_21	44.751	1.23E-40	CYP5068A2	26.18%	5.00E-38	Xenobiotic metabolism
SRUG_05169	estExt_fgenesh2_pg.C_10473	68.973	0	CYP504A7b	77.19%	0	Xenobiotic metabolism
SRUG_05987	e_gw1.28.62.1	83.902	0	CYP5080E1	44.58%	1.00E-105	Xenobiotic metabolism
SRUG_05824	estExt_Genewise1.C_500019	75.693	0	CYP5068B1	22.80%	6.00E-26	Xenobiotic metabolism
SRUG_06771	e_gw1.38.206.1	70.69	0	CYP53A8	84.60%	0	Xenobiotic metabolism
SRUG_06857	fgenesh1_kg.90_#_5_#_Locus1314v1rpkm98.06	69.929	0	CYP613B1c	99.61%	0	Xenobiotic metabolism
SRUG_07533	fgenesh1_kg.22_#_26_#_Locus5560v1rpkm19.37	62.548	0	CYP528A2	60.42%	1.00E-175	Xenobiotic metabolism
SRUG_07107	fgenesh1_kg.63_#_84_#_Locus2072v1rpkm61.91	73.823	0	CYP5078A4b	39.79%	6.00E-97	Xenobiotic metabolism
SRUG_08041	fgenesh1_kg.108_#_14_#_Locus442v2rpkm9.80_PRE	67.203	0	CYP530A1	89.46%	0	Xenobiotic metabolism
SRUG_09114	fgenesh1_kg.30_#_12_#_Locus7990v1rpkm8.36	61.155	0	CYP677A1	47.88%	1.00E-128	Xenobiotic metabolism
SRUG_09308	estExt_Genewise1.C_240063	60.179	0	CYP584E3b	27.06%	6.00E-34	Xenobiotic metabolism
SRUG_10249	CC1G_09160	51.83	0	CYP5068A1c	99.60%	0	Xenobiotic metabolism
SRUG_10264	MIX12671_3244_34	55.198	0	CYP53A7	31.34%	3.00E-24	Xenobiotic metabolism
SRUG_10626	fgenesh1_kg.153_#_19_#_Locus26056v3rpkm0.15	82.927	0	CYP532A6	35.46%	6.00E-79	Xenobiotic metabolism

SRUG_11224	fgenesh1_kg.23_#_203_#_Locus4066v1rpkm29.98	57.611	0	CYP677A1	47.88%	1.00E-128	Xenobiotic metabolism
SRUG_11583	MIX12671_3244_34	62.955	0	CYP53A7	31.34%	3.00E-24	Xenobiotic metabolism
